# Efficacy and safety of neoadjuvant PD-1 inhibitors or PD-L1 inhibitors for muscle invasive bladder cancer: a systematic review and meta-analysis

**DOI:** 10.3389/fimmu.2023.1332213

**Published:** 2024-01-09

**Authors:** Shibo Huang, Yanping Huang, Chunyan Li, Yiwen Liang, Miaoyan Huang, Raoshan Luo, Weiming Liang

**Affiliations:** The First Affiliated Hospital of Guangxi University of Science and Technology, Guangxi University of Science and Technology, Liuzhou, China

**Keywords:** PD-1 inhibitor, programmed cell death protein 1 inhibitor, programmed death-ligand 1 inhibitor, muscle invasive bladder cancer, neoadjuvant, complication

## Abstract

**Introduction:**

This meta-analysis aims to evaluate the efficacy and safety of neoadjuvant PD-1 inhibitors or PD-L1 inhibitors [PD-(L)1 inhibitors] for muscle-invasive bladder carcinoma (MIBC).

**Materials and methods:**

Four databases (Medline, Embase, Web of Science, and 21 CENTRAL) were searched for articles studying neoadjuvant PD-(L)1 inhibitors for MIBC. The search time period was from the establishment of each database to 21 July 2023. Meta-analyses of pCR, pPR, Grade≥ 3 irAEs rate, RFS, and OS were performed.

**Results:**

In total, 22 studies were included for meta-analysis. The overall pooled pCR of neoadjuvant PD-(L)1 inhibitors was 0.36 (95%CI=0.30–0.42, p=0.00). In subgroup meta-analysis, the pooled PCR of PD-(L)1 inhibitors alone, PD-(L)1 inhibitors plus other ICI, and PD-(L)1 inhibitors plus chemotherapy was 0.27 (95%CI=0.19–0.35, p=0.1), 0.41 (95%CI=0.21–0.62, p=0.01), 0.43 (95%CI=0.35–0.50, p=0.06), respectively. The overall pooled pPR of neoadjuvant PD-(L)1 inhibitors was 0.53 (95%CI=0.46–0.60, p=0.00). In subgroup meta-analysis, the pooled pPR of PD-(L)1 inhibitors alone, PD-(L)1 inhibitors plus other ICI, and PD-(L)1 inhibitors plus chemotherapy was 0.36 (95%CI=0.22–0.51, p=0.01), 0.51 (95%CI=0.39–0.62, p=0.43), and 0.61 (95%CI=0.53–0.69, p=0.01), respectively. Kaplan–Meier curves for OS and RFS were reconstructed, but there was no significant difference among three groups in terms of OS or RFS. The pooled result of Grade≥ 3 irAEs rate for neoadjuvant PD-(L)1 inhibitors was 0.15 (95%CI=0.09–0.22, p=0.00%). In subgroup analysis, the pooled result of Grade≥ 3 irAEs rate for PD-(L)1 inhibitors alone, PD-(L)1 inhibitors plus other ICI, and PD-(L)1 inhibitors plus chemotherapy was 0.07 (95%CI=0.04–0.11, p=0.84), 0.31 (95%CI=0.16–0.47, p=0.06), and 0.17 (95%CI=0.06–0.31, I^2^ = 71.27%, p=0.01), respectively.

**Conclusion:**

Neoadjuvant PD-(L)1 inhibitors were feasible and safe for muscle invasive bladder cancer. Compared with PD-(L)1 inhibitors alone, PD-(L)1 inhibitors plus other ICI and PD-(L)1 inhibitors plus chemotherapy were associated with higher pCR and pPR, but higher Grade≥3 irAEs. Kaplan–Meier curves for OS and RFS indicated that neoadjuvant PD-(L)1 inhibitors had an acceptable long-term prognostic, but it was not possible to discern statistical differences between the three neoadjuvant subgroups.

**Systematic review registration:**

https://www.crd.york.ac.uk/prospero/display_record.php?ID=CRD42023452437, identifier PROSPERO (CRD42023452437).

## 1 Introduction

Bladder cancer is the most common malignancy of the urinary system with high prevalence in the world (1). Approximately 30% of bladder cancers are muscle-invasive bladder carcinoma (MIBC), which are related to high risk of metastases-related death, and another 70% of bladder cancers are non-muscle-invasive bladder carcinoma (NMIBC), which is not as serious as MIBC (2). According to the risk stratification of the European Association of Urology (EAU) guidelines, NMIBC can be further classified as low-, intermediate-, and high-risk groups based on risk of recurrence and/or progression (3). Unfortunately, 60%–80% of patients with high-risk NMIBC would have a relapse, and 20%–40% of them would develop into MIBC after 5 years (4–6). The prognosis of MIBC remains poor, with the 5-year overall survival (OS) rate decreasing to 60% (7).

Neoadjuvant chemotherapy (NAC) followed by radical cystectomy (RC) has been recommended for eligible patients with MIBC (8, 9). Commonly used chemotherapy regimens are platinum-based NACs, including gemcitabine and cisplatin (GC), and dose-dense methotrexate, vinblastine, doxorubicin, and cisplatin (ddMVAC) (10, 11). NAC has obviously improved the OS of MIBC, with the 5-year OS rate approaching 90% for patients achieving a pathological partial response (pPR) at the time of RC (12). However, NAC reported frequent adverse events (AEs), and a partial of cisplatin-eligible MIBC patients have to discontinue the treatment protocol because of severe treatment-related adverse (10, 13). In addition, NAC cannot meet the needs of cisplatin-ineligible patients with MIBC (14). Thus, alternative treatment options are highly necessary.

Recently, the use of immune checkpoint inhibitors (ICIs) has reshaped the treatment paradigm and revolutionized the prognosis of several cancers, such as non-small-cell lung cancer, melanoma, and renal cell carcinoma (15–18). Antibodies against programmed cell death 1 or its ligand have been used for the treatment of advanced/metastatic urothelial cancer, and a significant clinical benefit of PD-(L)1 has been demonstrated (19, 20). At the same time, a growing number of multiple clinical trials have explored combination of PD-(L)1 inhibitors and platinum-based chemotherapy with the reduced risk of developing resistance and/or anticipation of synergistic effect (21, 22). Considering the effectiveness of PD-(L)1 inhibitors in metastatic bladder cancer, clinical trials have been developed to explored the feasibility and safety of neoadjuvant therapy using PD-(L)1 inhibitors (23–25). Basile et al. reported a 37% pathological complete response (pCR) rate and 55% pathological partial response(pPR) rate in the PURE-01 study in which three cycles of pembrolizumab were given to patients with a diagnosis of MIBC and eligible for RC, and 36-month event-free survival (EFS) and over survival (OS) were 74.4% and 83.8% (24, 26, 27). Other clinical trials have been conducted to evaluate the safety and efficacy of PD-(L)1 inhibitors combined with chemotherapy or PD-(L)1 inhibitors combined with other ICI strategies. Kim et al. reported a 35% pCR rate of RC patients after neoadjuvant nivolumab plus gemcitabine/cisplatin chemotherapy (28). The NABUCCO study investigating ipilimumab plus nivolumab reported a 45.8% pCR rate (29).

In the present study, we aimed to systematically assess the available evidence in the literature regarding the safety and efficacy of neoadjuvant PD-(L)1 inhibitors in patients with stage II–III MIBC.

## 2 Materials and methods

### 2.1 Search strategy

The present meta-analysis was conducted according to the Preferred Reporting Project for Systematic Review and Meta-Analysis (PRISMA) 2020 guidelines. This study has been registered at PROSPERO with a registration number of CRD42023452437. Four databases including PubMed, Embase, Web of Science, and the Cochrane Library were systematically searched for literatures published up to 21 July 2023, using the following searching strategy: (“PD-1 inhibitor” OR “PD-L1 inhibitor”) AND “neoadjuvant” AND “bladder cancer” AND (“randomized controlled trial” OR “prospective” OR “retrospective”). **Supplementary Material 1** presents the searching record in detail.

### 2.2 Inclusion and exclusion criteria

Inclusion criteria were as follows: (1) patients diagnosed as MIBC (stage II/III); (2) neoadjuvant therapy using PD-(L)1 inhibitors was administrated, with or without chemotherapy or other ICI, and RC was performed after neoadjuvant therapy; (3) at least one of the following outcomes were reported, namely, pCR, pPR, OS, RFS, Grade≥ 3 irAEs rate, Grade≥ 3 TRAEs rate; and s(4) study types, namely, randomized controlled studies, non-randomized controlled studies, single-arm trials, prospective studies, and retrospective studies.

Exclusion criteria were as follows: (1) other types of articles, such as case reports, publications, letters, reviews, meta-analyses, editorials, pharmacological intervention, animal studies, and protocols; (3) other cancers; (4) no relative outcomes; (5) reduplicate cohort of patients; and (6) failure to extract data for meta-analysis.

### 2.3 Data extraction

Two independent investigators (S.H. and Y.H.) reviewed the title and abstract and then read the full text. Discrepancy were resolved by consulting with a third investigator (M.H.). Data retrieved included first author’s name, year, trial ID, study design, sample size, intervention, male ratio, age, study design, cTNM stage, cisplatin eligibility, regimen, pCR, pPR, OS, RFS, Grade≥ 3 irAEs rate, Grade≥ 3 TRAEs rate, Kaplan–Meier curves for OS, and Kaplan–Meier curves for RFS.

### 2.4 Risk of bias assessment

The risk of bias was assessed by two independent reviewers (L.H. and S.H.), using the modified Jadad scale (30) for RCTs while using the methodological index for non-randomized studies (MINORS) (31) for single-arm studies or non-RCTs.

### 2.5 Statistical analysis

The selection duplicate removal of studies included was conducted using EndNote (Version 20; Clarivate Analytics). All analyses were performed using Stata 12.0 and R version 4.3.1 [R version Copyright (C) 2023, The R Foundation for Statistical Computing]. The “meta” package and IPDformKM package were utilized in the analysis. GetData Graph Digitizer software was used to extract data from articles containing Kaplan–Meier curves, and individual data were reconstructed with IPDformKM package. The established method by Guyot et al. was used to reconstruct individual patient-level data (32). Continuous variables were compared using weighted mean difference (WMD) with a 95% confidence interval (CI). Relative ratio (RR) with 95% CI were used to compare binary variables. The medians and interquartile ranges of continuous data were converted to the mean and standard deviation. Statistical heterogeneity between included studies was calculated using the Cochrane ‘Sq test and the I^2^ index (I^2^ >50% indicating high heterogeneity). When there is high heterogeneity among studies, the random effects model is adopted, otherwise the fixed effects model is adopted (33). A p-value < 0.05 was considered statistically significant. Begg’s method was used to test the publication bias among various studies and to draw a funnel plot. Finally, a sensitivity analysis was performed to determine the impact of individual studies on the aggregated results and to test the reliability of the results.

## 3 Results

### 3.1 Search results

**Figure 1:**
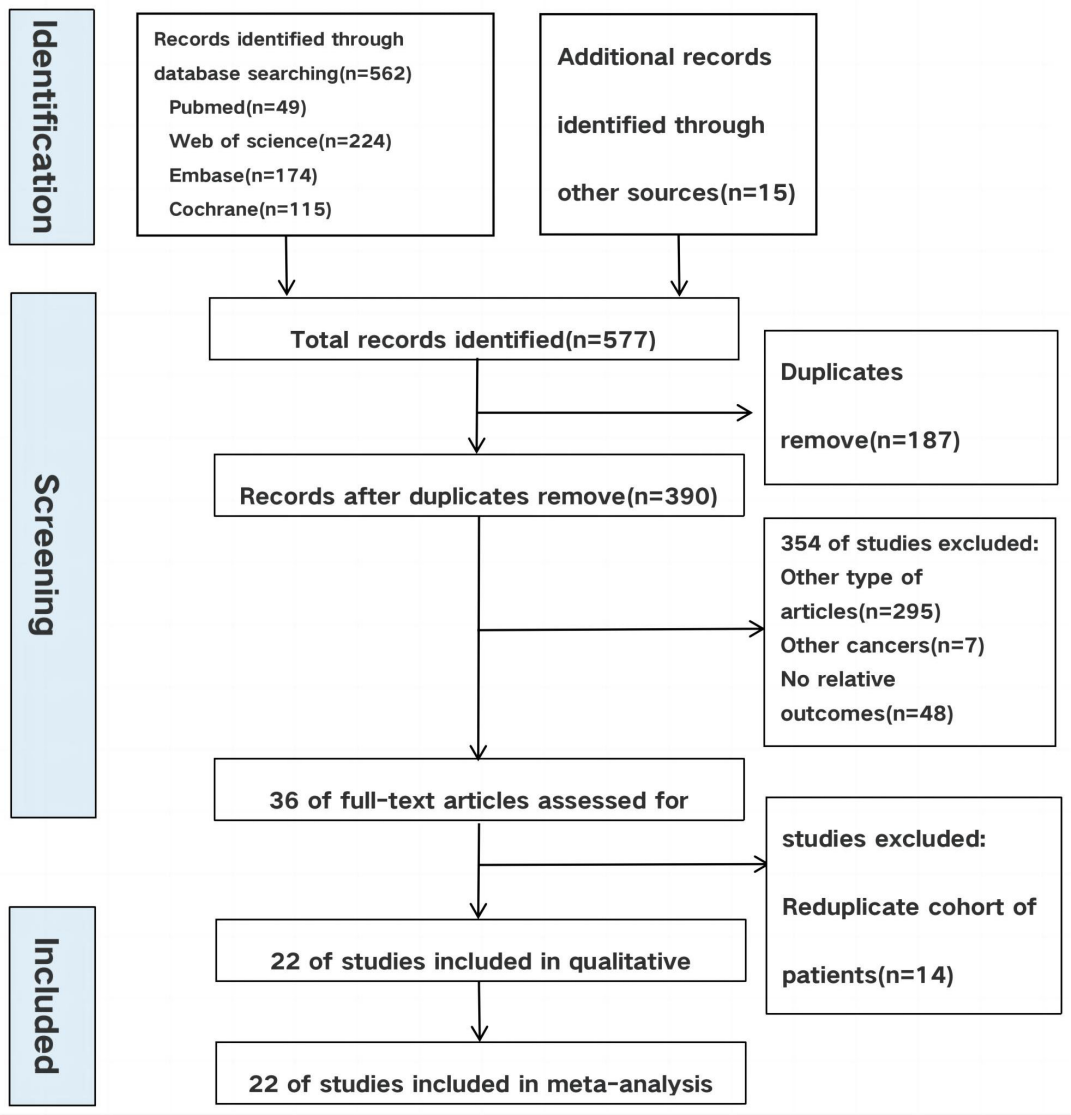
Flow chart of literature search strategies.

The process of the literature selection and inclusion is presented in **Figure 1**. Our initial search found a total of 577 studies. After excluding repeat studies, only 390 cases remained. By reading the full text, 295 other types of articles, 7 articles investigating other types of cancer, and 48 unrelated articles were excluded. Finally, 22 studies involving 843 patients with advanced bladder cancer were ultimately included in this meta-analysis.

### 3.2 Patient characteristics and quality assessment

Most of the included studies were phase II single-arm trials with a total of 22 cohorts, eight of which explored neoadjuvant PD-(L)1 inhibitors alone (two pembrolizumab (27, 34), two atezolizumab (35, 36), two nivolumab (37, 38), one durvalumab (39), and one avelumab (40)), five cohorts exploring PD-(L)1 inhibitors plus other ICI (three ipilimumab plus nivolumab (29, 37, 41) and two durvalumab plus tremelimumab (42, 43)), and PD-(L)1 inhibitors plus chemotherapy in 11 cohorts (eight gemcitabine/cisplatin [GC] plus ICI (28, 44–50), one dose-dense course of methotrexate, vinblastine, doxorubicin, and cisplatin [ddMVAC] plus ICIs (51), one gemcitabine plus ICI (52), and one paclitaxel/gemcitabine [PG] plus ICI) (40). The quality of RCT literature was evaluated using modified Jadad scale for RCTs, and both RCTs were high-quality articles. Other articles were scored using MINORS, with 15 points for 4 articles, 14 points for 8 articles, 13 points for 2 articles, 12 points for 5 articles, and 6 points for 2 articles. A total of 13 cases were recorded involving 542 patients, and the proportion of TNM stages was reported in detail: 65.7% for cT2, 33.4% for cT3-4a, and 2.0% for cN1. Details of all studies and the characteristics of the patients with bladder cancer are shown in **Table 1**.

**Table 1:** Characteristics of included studies and patients.

Hor	Registration ID	Year	Study design	cTNM stage	Cis-ineligible or refusal	Study arm(s)	No. of patients	Regimen, cycles	Age (median, years)	Gender (male, %)	Quality
Kim (28)	KCT0003804 CRIS	2022	single-arm	T2-4aN0M0	No	GC+ Nivolumab	51	3–4	NA	NA	14
Van Dijk (29)	NCT03387761cohort I	2020	single-arm	T2-T4aN0-1M0,	Regardless	Nivolumab + Ipilimumab	24	3	65	75%	15
Goubet (34)	NCT03212651	2022	single-arm	T2-4aN0M0	NA	Pembrolizumab	39	3	NA	NA	12
Necchi (27)	NCT02736266	2022	single-arm	T2-4aN0M0	Regardless	Pembrolizumab	114	3	66	86.8%	15
Szabados (35)	NCT02662309	2022	single-arm	T2–T4aN0M0	Yes	Atezolizumab	95	2	73	85%	15
Koshkin (36)	NCT02451423	2021	single-arm	T2-4aN0-1M0	Yes	Atezolizumab	20	1-3	69	75%	14
Guercio (37)	NCT03520491	2022	non-RCT	T2-4aN0M0,	Yes	armA: Nivolumab	armA:15	NA	76	80%	13
						armB: Nivolumab + Ipilimumab	armB: 15				
Yin (38)	NCT03532451	2021	non-RCT	T2-4aN0-1M0	Yes	armA: Nivolumab	armA:13	NA	75	67%	14
Wei (39)	NCT03773666	2020	single-arm	T2-4aN0M0	Yes	Durvalumab	10	3	67	80%	14
Chanza (40)	NCT03674424	2022	RCT	T2-4aN0-1M0	armA: No	armA: PG+ Avelumab	armA:28	4	armA: 72	armA: 93%	6
					armB: Yes	armB: Avelumab	armB: 28		armB: 75	armB: 93%	
Van Dorp (41)	NCT03387761cohort II	2021	single-arm	stage III	Yea	Nivolumab + Ipilimumab	30	3	NA	NA	13
Grande (42)	NCT03472274	2020	RCT	cT2‐4aN0-1M0	No	armA: Durvalumab+Tremelimumab	armA:23	3	NA	NA	6
						armB: GC/ddMVAC	armB: 38				
Gao (43)	NCT02812420	2020	single-arm	T2-4aN0M0	Yes	Durvalumab+ Tremelimumab	28	2	71	71%	15
Xing (44)	ChiCTR2000032359	2023	single-arm	T2-4aN0-1M0	No	GC+ Camrelizumab	19	3	69	73.7%	12
Rose (45)	NCT02690558	2021	single-arm	T2-4aN0-1M0	No	GC+ Pembrolizumab	39	4	NA	NA	14
Lin (46)	ChiCTR2000037670	2022	single-arm	T2-4aN0M0	No	GC+ Tislelizumab	17	4	62	NA	12
Kaimakliotis (47)	NCT02365766	2019	single-arm	T2-4aN0M0	No	GC+ Pembrolizumab	40	4	65	75%	14
Gupta (48)	NCT03294304	2022	single-arm	T2-4aN0-1M0	No	GC+ Nivolumab	41	4	NA	NA	14
Funt (49)	NCT02989584	2021	single-arm	T2-4aN0M0	No	GC+ Atezolizumab	44	4	NA	NA	12
Cathomas (50)	SAKK 06/17	2020	single-arm	T2-4aN0-1M0	Yes	GC+ Durvalumab	61	4	67.5	79%	14
Thibault (51)	NCT03549715	2020	single-arm	NA	No	ddMVAC+ Durvalumab+Tremelimumab	12	2	59.5		12
Hristos (52)	NCT02365766 cohort2	2020	single-arm	T2-4aN0M0	Yes	Gemcitabine+Pembrolizumab	37	3	72	70%	13

### 3.3 pCR

**Figure 2:**
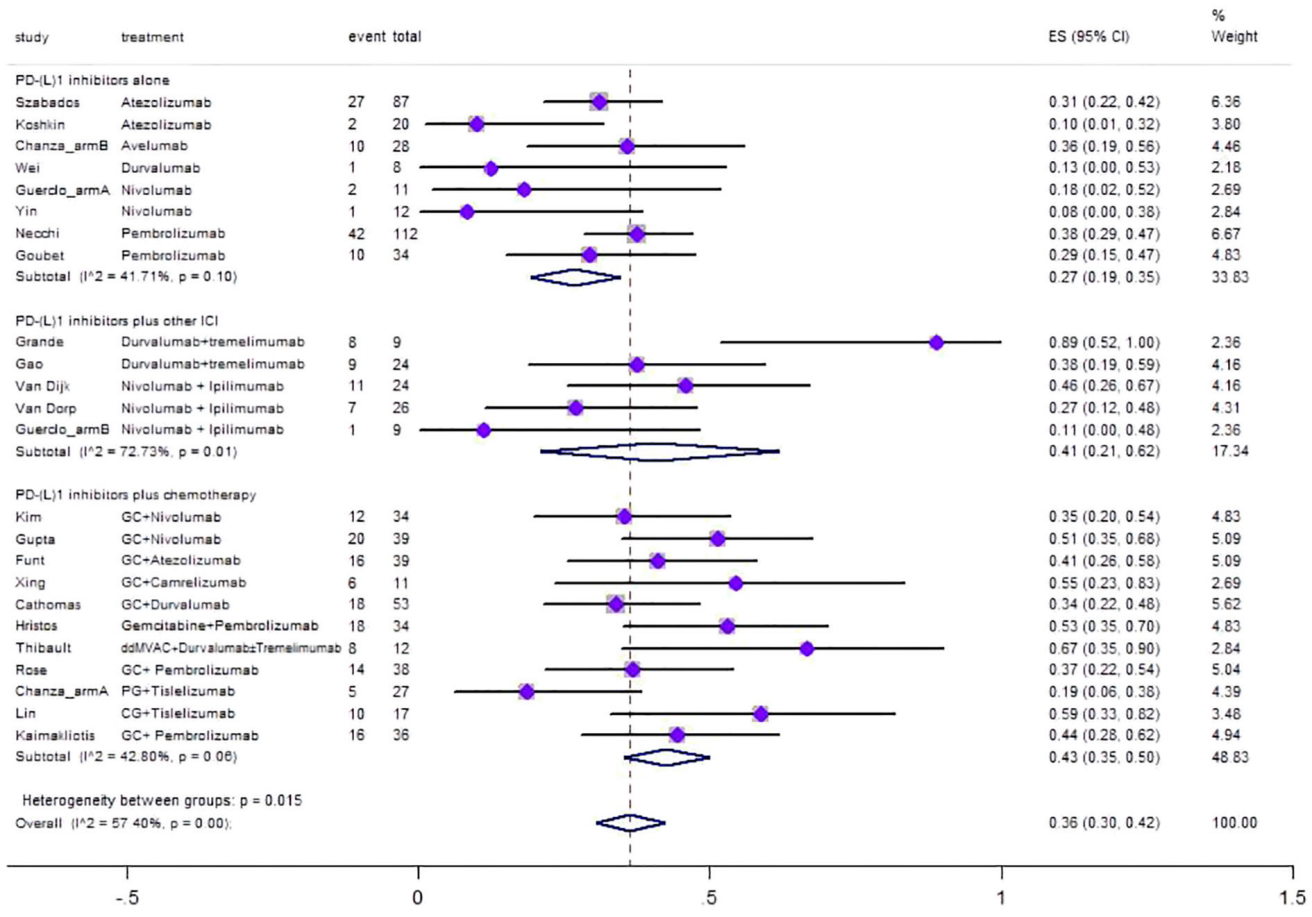
Forest plot of the meta-analysis for pCR.

**Figure 2** shows forest plot of the meta-analysis for pCR. The overall pooled pCR of neoadjuvant PD-(L)1 inhibitors was 0.36(95%CI=0.30–0.42, I^2 = ^57.4%, p=0.00). Results of subgroup meta-analysis are shown in **Table 2**.

**Table 2:** Results of the meta-analysis for pCR, pPR, and Grade≥ 3 irAEs rate.

Outcomes	No. of studies	Heterogeneity	Overall effect size	95% CI of overall effect	Weight(%)
		I^2^(%) p-value			
PCR					
**PD-(L)1 inhibitors alone**	**8**	**41.71 0.10**	**0.27**	**0.19–0.35**	**33.83**
**PD-(L)1 inhibitors plus other ICI**	**5**	**72.73 0.01**	**0.41**	**0.21–0.62**	**17.34**
**PD-(L)1 inhibitors plus chemotherapy**	**11**	**42.80 0.06**	**0.43**	**0.35–0.50**	**48.83**
**Overall pooled PCR**	**24**	**57.40 0.00**	**0.36**	**0.30–0.42**	**100**
PPR					
**PD-(L)1 inhibitors alone**	**6**	**65.79 0.01**	**0.36**	**0.22–0.51**	**26.26**
**PD-(L)1 inhibitors plus other ICI**	**4**	**0.00 0.43**	**0.51**	**0.39–0.62**	**17.56**
**PD-(L)1 inhibitors plus chemotherapy**	**11**	**55.15 0.01**	**0.61**	**0.53–0.69**	**56.19**
**Overall pooled PPR**	**21**	**60.94 0.00**	**0.53**	**0.46–0.60**	**100**
Grade≥ 3 irAEs rate					
**PD-(L)1 inhibitors alone**	**7**	**0.00 0.84**	**0.07**	**0.04–0.11**	**44.05**
**PD-(L)1 inhibitors plus other ICI**	**4**	**59.17 0.06**	**0.31**	**0.16–0.47**	**24.36**
**PD-(L)1 inhibitors plus chemotherapy**	**5**	**71.27 0.01**	**0.17**	**0.06–0.31**	**31.59**
**Overall pooled Grade≥ 3 irAEs rate**	**16**	**69.83 0.00**	**0.15**	**0.09–0.22**	**100**

### 3.4 pPR

**Figure 3:**
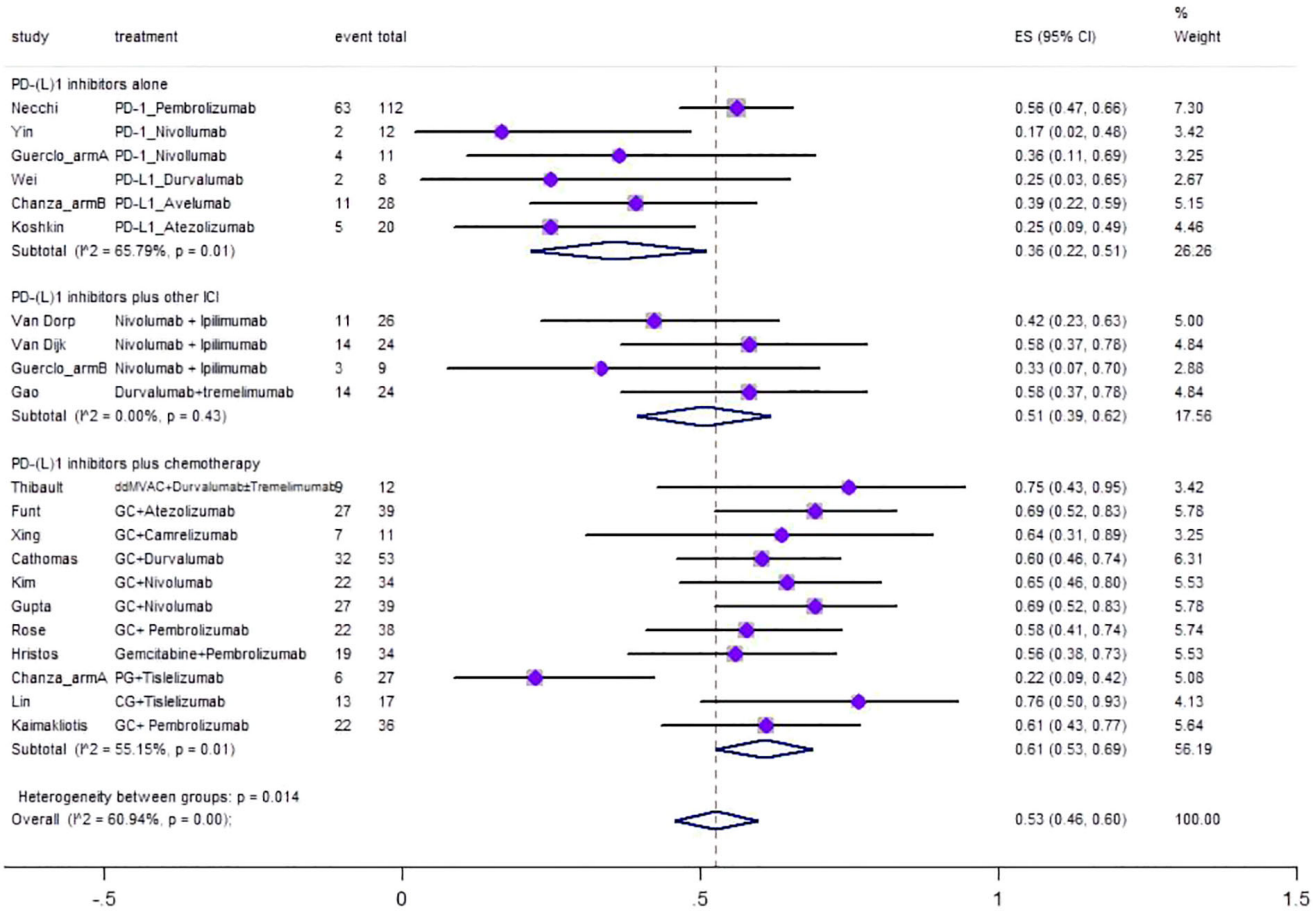
Forest plot of the meta-analysis for pPR.

**Figure 3** shows the forest plot of the meta-analysis for pPR. The overall pooled pPR of neoadjuvant PD-(L)1 inhibitors was 0.53 (95%CI=0.46–0.60, I^2 = ^60.94%, p=0.00). Results of subgroup meta-analysis are shown in **Table 2**.

### 3.5 OS

**Figure 4:**
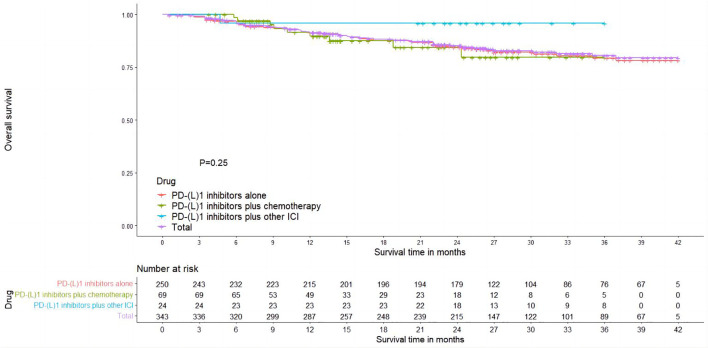
Kaplan–Meier curves for OS.

In total, five studies reported Kaplan–Meier curves for overall survival (OS), with two studies reporting PD-(L)1 inhibitors plus chemotherapy (49, 53), two studies reporting PD-(L)1 inhibitors alone (26, 54), and one study reporting PD-(L)1 inhibitors plus other ICI (55). Using the IPDformKM package, we extracted individual data and reconstructed Kaplan–Meier curves for OS (**Figure 4**). The OS of neoadjuvant PD-(L)1 inhibitors was 91.67%, 86.03%, and 81.64% at 1 year, 2 years, and 3 years, respectively. Results of subgroup meta-analysis are shown in **Table 3**. However, there was no significant difference in OS among the three groups (p=0.25).

**Table 3:** Results of OS and RFS.

Outcomes	No. Of studies	1 year	2 years	3 years
OS				
**PD-(L)1 inhibitors alone**	**2**	**91.7%**	**84.85%**	**80.28%**
**PD-(L)1 inhibitors plus other ICI**	**1**	**96.05%**	**96.05%**	**96.05%**
**PD-(L)1 inhibitors plus chemotherapy**	**2**	**92.11%**	**80.07%**	**80.07%**
**Neoadjuvant PD-(L)1 inhibitors**	**5**	**91.67%**	**86.03%**	**81.64%**
RFS				
**PD-(L)1 inhibitors alone**	**2**	**85.3%**	**80.12%**	**79.3%**
**PD-(L)1 inhibitors plus other ICI**	**1**	**91.72%**	**91.72%**	**91.72%**
**PD-(L)1 inhibitors plus chemotherapy**	**3**	**84.47%**	**71.84%**	**71.84%**
**Neoadjuvant PD-(L)1 inhibitors**	**6**	**85.69%**	**79.67%**	**79.05%**

### 3.6 RFS

**Figure 5:**
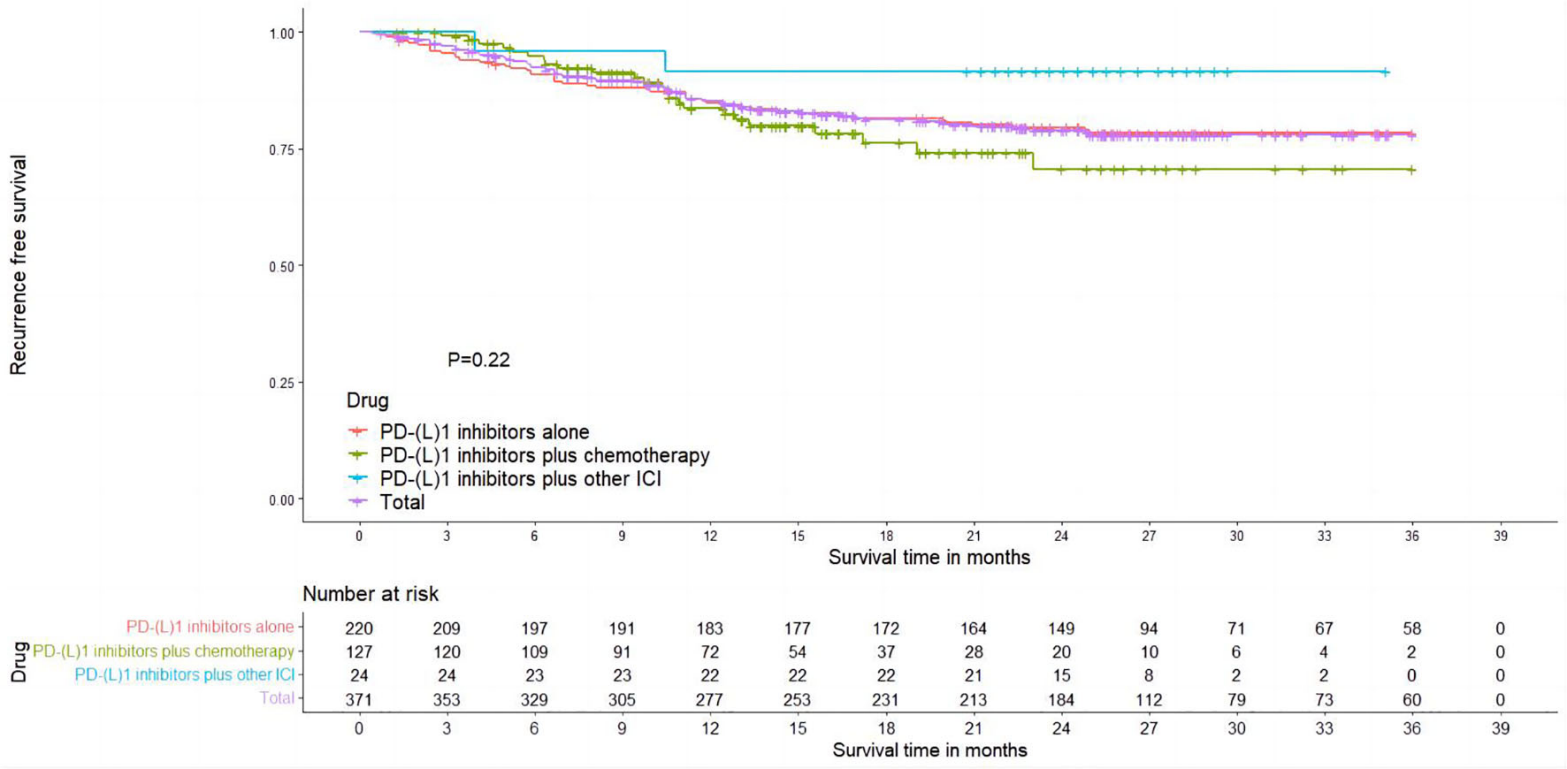
Kaplan–Meier curves for RFS.

Totally, six studies reported Kaplan–Meier curves for recurrence-free survival (RFS), with three studies reporting PD-(L)1 inhibitors plus chemotherapy (28, 45, 49), two studies reporting PD-(L)1 inhibitors alone (26, 54), and one study reporting PD-(L)1 inhibitors plus other ICI (55). Using the IPDformKM package, we extracted individual data and reconstructed Kaplan–Meier curves for RFS (**Figure 5**). The RFS of neoadjuvant PD-(L)1 inhibitors was 85.69%, 79.67%, and 79.05% at 1 year, 2 years, and 3 years, respectively. Results of subgroup meta-analysis are shown in **Table 3**. However, there was no significant difference in RFS among the three groups (p=0.22).

### 3.7 Safety

**Figure 6:**
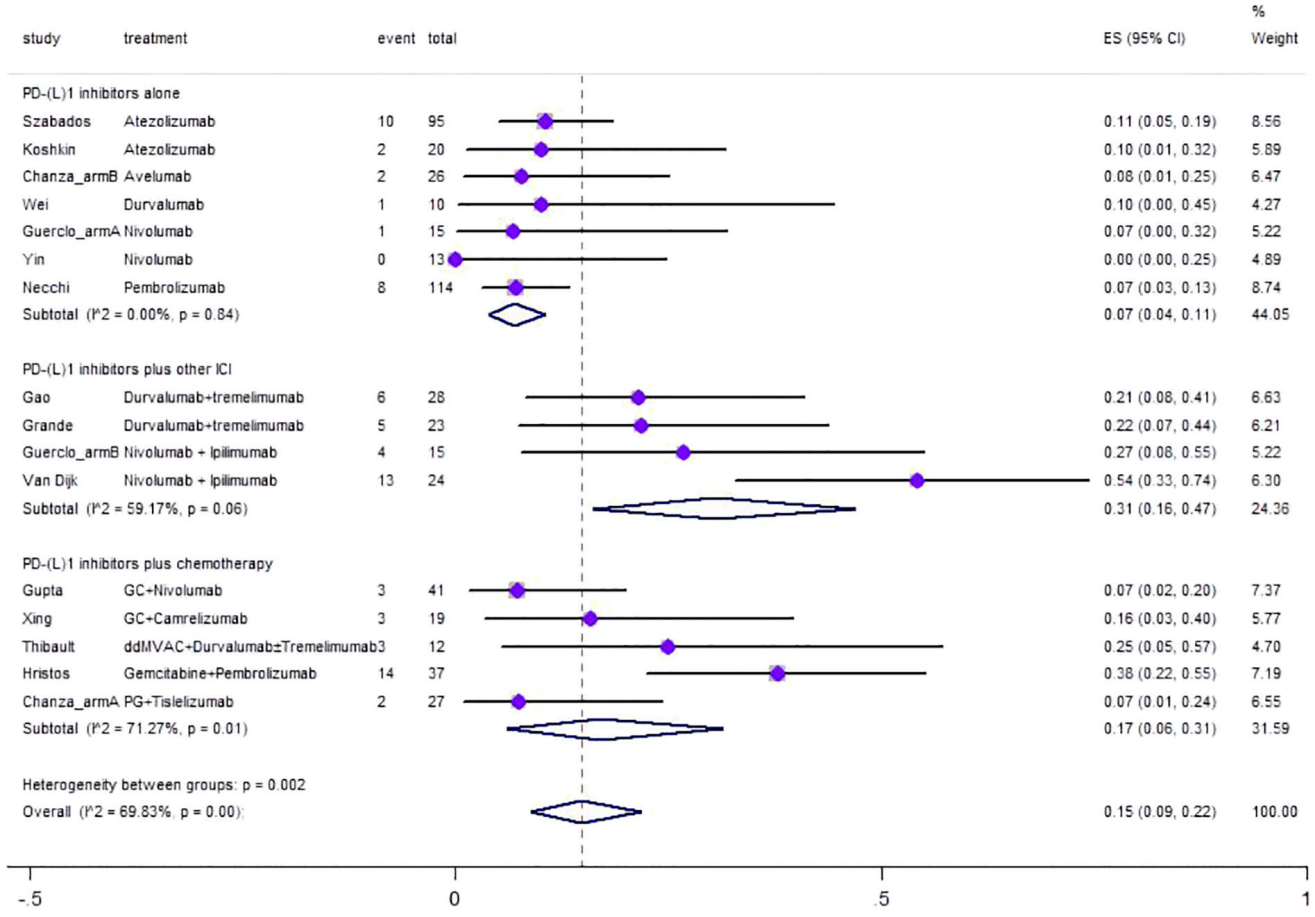
Forest plot of the meta-analysis for Grade≥ 3 irAEs rate.

Regarding safety, Grade≥ 3 irAEs rate was evaluated, which was reported in a total of 17 cohorts (**Figure 6**). The pooled result of Grade≥ 3 irAEs rate for neoadjuvant PD-(L)1 inhibitors was 0.15 (95%CI=0.09–0.22, I^2 = ^69.83%, p=0.00). Results of subgroup meta-analysis are shown in **Table 2**. The common irAEs included elevated liver enzymes, elevated amylase/lipase, imDC, hematological toxicity, skin reactions, and fatigue.

### 3.8 Supplement oncological and safety outcomes

**Supplementary Material 2** reports PCR (%), PRR (n), ≥ Grade3 irAEs, ≥ Grade 3 surgical complications, and AEs in detail.

## 4 Discussion

Since the significant clinical benefit of PD-(L)1 inhibitors demonstrated in patients with advanced/metastatic urothelial cancer, a growing number of clinical trials has been performed to evaluate the safety and efficacy of PD-(L)1 inhibitors in the neoadjuvant therapy for MIBC patients. In these clinical trials, PD-(L)1 inhibitors were used alone, combined with chemotherapy, or combined with other ICIs. In the present study, a systemic review and meta-analysis was conducted to evaluate the safety and efficacy of neoadjuvant PD-(L)1 inhibitors in patients with MIBC.

PD-(L)1 inhibitors alone or plus other ICI, PD-(L)1 inhibitors provided an optional treatment modality for patients who either were ineligible or refused cisplatin-based neoadjuvant chemotherapy. PD-(L)1 inhibitors plus other ICI seem to have advantage in efficacy over PD-(L)1 inhibitors alone. In the present study, the polled analysis showed that pCR of PD-(L)1 inhibitors plus other ICI was higher than that of PD-(L)1 inhibitors alone, and similar results were present regarding pRR. However, our study showed that there was no significant difference among three groups in terms of OS or RFS. Only five studies reported Kaplan–Meier curves for OS, and six studies reported Kaplan–Meier curves for RFS, with relatively short follow-up time. The statistical results of oncology outcomes were difficult to reflect the differences among three groups due to the small sample and short follow-up time. In previous literature, PD-(L)1 inhibitors were effective in the neoadjuvant therapy for non-small-cell lung cancer (NSCLC). Forde et al. conducted a phase 2 study designed to evaluate the safety and feasibility of administration of two doses of nivolumab over 4 weeks before surgery in patients with stage I–IIIA resectable NSCLC and reported a major pathological response rate of 45% with a complete pathological response rate of 10% (56). Although median DFS and OS have not yet been reached in this study, 80% of patients were alive without recurrence at 1 year. Recent clinical trials have declared the safety, feasibility, and efficacy of neoadjuvant PD-(L)1 inhibitors in solid tumors other than MIBC, including triple-negative breast cancer, melanoma, and NSCLC (57–59).

The administration of immune checkpoint inhibitors in the neoadjuvant therapy has several advantages (60). First, with neoadjuvant therapy of immune checkpoint inhibitors, the intact tumor could become the source for antigen-specific T-cell immunity with multiple antigen load. Second, the early evaluation of therapy response in individual patients by pathological analysis on the excised tumor allows for potential to adjust systemic therapy according to pathological response. Furthermore, a unique platform for relative basic and translational investigations can be provided by neoadjuvant therapy strategies with immune checkpoint inhibitors (61).

Liu et al. used two models of spontaneously metastatic breast cancers in mice to illustrate the significantly therapeutic power of neoadjuvant in the context of primary tumor resection and found that mice treated with anti-PD-1/anti-CD137 combination before surgery demonstrated a 40% long-term survival compared with 0% in the adjuvant group (62). In addition, an increase in tumor-specific CD8+ T cells was seen in the neoadjuvant group but not in the adjuvant group, which suggested that neoadjuvant ICIs with the tumor *in situ* contribute to a more robust T-cell response. This study highlighted the above advantages of neoadjuvant therapy with immune checkpoint inhibitors.

Regarding safety, irAEs seem to occur more frequently when PD-(L)1 inhibitors plus other ICI were administrated. In the present study, Grade≥ 3 irAEs morbidity was 0.51 in patients who were treated by PD-(L)1 inhibitors plus other ICI, while the rate was 0.36 in PD-(L)1 inhibitors alone group. Similarly, a randomized, open-label, multicenter, phase 3 trial (DANUBE) in patients with untreated, unresectable, locally advanced, or metastatic urothelial carcinoma reported that grade 3 or 4 treatment-related adverse events occurred in 47 (14%) of 345 patients in the durvalumab group while 93 (27%) of 340 patients in the durvalumab plus tremelimumab group (63). Thus, the safety profile should not be ignored when PD-(L)1 inhibitors plus other ICI were administrated.

Although NAC has been preferred by the National Comprehensive Cancer Network, only 36%–49% of MIBC patients treated by NAC can achieve non-muscle invasive downstaging (13, 64). A more effective neoadjuvant therapy is urgent for patients with MIBC. Several clinical trials has reported the efficacy of PD-(L)1 inhibitors in the treatment of platinum-resistant metastatic bladder carcinoma, which demonstrated that there is no clinical cross-resistance between NAC and PD-(L)1 inhibitors (65–67). Recent studies reported that PD-(L)1 inhibitors plus chemotherapy resulted in better RFS and OS in patients with advanced or metastatic MIBC, compared with chemotherapy alone (21, 68). Based on the above results, several clinical trials have recently been conducted to assess the efficacy of neoadjuvant PD-(L)1 inhibitors plus chemotherapy for patients with MIBC. The pooled result of the present meta-analysis showed that the pCR and pPR was 43% and 61% for neoadjuvant PD-(L)1 inhibitors plus chemotherapy, respectively, which seems to have advantage over NAC in oncological outcomes. A meta-analysis comparing oncological outcomes of ddMVAC with GC as neoadjuvant chemotherapy for muscle-invasive bladder cancer reported a pCR of 35.2% in patients treated by ddMVAC while 25.1% in patients treated by GC[50]. A recent randomized phase III trial comparing dd-MVAC with GC reported that pCR was observed in 42% of the ddMVAC group and in 36% of the GC group, respectively, and <pT2N0 rates of 63% and 49%[51]. A retrospective study reported that the mean Kaplan–Meier estimates of OS was 4.2 years in the GC group and 7.0 years in the ddMVAC group (69). A cross-sectional analysis indicated that 2-year Kaplan–Meier survival probability estimates were 73.3% for ddMVAC and 62% for GC (70). Therefore, compared with NAC alone, neoadjuvant PD-(L)1 inhibitors plus chemotherapy provided a more effective treatment modality for patients who were fit for cisplatin-based neoadjuvant chemotherapy. There is an important question that needs to be answered: is there a major advantage of the use of PD-(L)1 inhibitors over neoadjuvant cisplatin-based chemotherapy? In view of the revolution brought about by the EV 302 trial (71), the KEYNOTE-B15/EV-304 (NCT04700124) trial is now underway, which is a phase 3 trial that aims to assess the effectiveness and safety of perioperative Enfortumab vedotin (EV) plus pembrolizumab compared to neoadjuvant chemotherapy using gemcitabine/cisplatin in patients with muscle-invasive bladder cancer who are eligible for cisplatin treatment (72). The outcome of this trial is eagerly awaited to answer the above question.

Regarding safety, our results showed that the Grade≥ 3 irAEs rate was 17% after neoadjuvant PD-(L)1 inhibitors plus chemotherapy, while the Grade≥ 3 TRAEs rate was 47%. A retrospective multicenter study of a clinical database reported that the Grade≥ 3 AEs occurred in 31% patients during neoadjuvant chemotherapy for muscle invasive bladder cancer (73). A recent randomized trial reported that 52% patients had Grade≥ 3 AEs in dd-MVAC arm while 55% in GC arm (13).

In light of the potential significant negative consequences, the high expenses associated with therapy, and the emergence of alternative therapeutic options, the significance of predictive biomarkers for personalized treatment seems more crucial than ever. Several trials included in the study evaluated PD-L1 testing and the rate of positivity, and the secondary endpoints of these trials reported the pCR rate in patients who tested positive for PD-L1 (27, 29, 35, 46, 47). In the ABACUS trial, Thomas Powles et al. characterized PD-L1 positivity as the presence of ≥5% of immune cells staining using the SP142 antibody. However, some other trials have classified PD-L1 positivity as CPS>10%. Three trials demonstrated no statistically significant differences in pCR rates between patients who tested positive for PD-L1 and those who tested negative for PD-L1 (29, 35, 46). Nevertheless, the PURE-01 research found that PD-L1 positivity (OR, 1.02; 95% CI, 1.01–1.04) was a statistically significant factor (27). This suggests that the presence of PD-L1 could potentially be used to predict the response to PD-(L)1 inhibitors in terms of pathology. In addition, tumor mutational burden (TMB) enhances the amount of tumor neoantigens and the likelihood of effective T-cell identification. The field of urothelial carcinoma (UC) has observed noteworthy correlations between elevated tumor mutational burden (TMB) and positive treatment outcomes in both the neoadjuvant therapy context (PURE-01 trial) (27) and for metastatic tumors (IMvigor210, KEYNOTE-028) (74, 75). Circulating tumor DNA (ctDNA) refers to bits of DNA from tumors that are present in the bloodstream. It has been discovered that only patients who test positive for ctDNA receive a significant advantage from adjuvant atezolizumab treatment (IMvigor010), indicating that ctDNA can be used to identify individuals at a high risk of metastasis in UC. Currently, there are ongoing clinical trials (TOMBOLA, IMvigor011) that are enrolling patients who have detectable ctDNA following radical cystectomy for the purpose of receiving atezolizumab treatment. However, in this instance, ctDNA functions as a prognostic biomarker rather than a predictive one (71, 76, 77).

There were several strengths in the present study. First of all, few meta-analysis has assessed the efficacy and safety of PD-(L)1 inhibitors in the neoadjuvant therapy for MIBC, and we conducted a systemic review and meta-analysis including the latest studies on neoadjuvant PD-(L)1 inhibitors in patients with stage II–III MIBC. Second, the outcomes were pooled by PP and subgroup analyses, since discrepancies of different literature were included. Third, Kaplan–Meier curves for OS and RFS were reconstructed using the IPDformKM package, presenting an intuitive impression for oncological outcomes. Specifically, three protocols were analyzed: PD-(L)1 inhibitors alone, PD-(L)1 inhibitors plus other ICI, and PD-(L)1 inhibitors plus chemotherapy. The PP analysis contributes to represent the latest progress of each treatment regimens.

Our study has several limitations. First of all, most studies were non-randomized single-arm clinical trials with a small sample size, resulting in indirect comparisons among different treatment regimens. Second, there was significant heterogeneity in the majority of clinical outcomes. The probable reasons consist of the included population bias and the difference in drug types, dosage, and cycles of regimens. Third, most studies have not yet reached their endpoint, which failed to provide data of survival outcomes, making it difficult to assess the lasting benefits of neoadjuvant PD-(L)1 inhibitors.

In conclusion, neoadjuvant PD-(L)1 inhibitors were feasible and safe for muscle-invasive bladder cancer. Compared with PD-(L)1 inhibitors alone, PD-(L)1 inhibitors plus other ICI and PD-(L)1 inhibitors plus chemotherapy were associated with higher pCR and pPR but higher Grade≥ 3 irAEs. Kaplan–Meier curves for OS and RFS indicated that neoadjuvant PD-(L)1 inhibitors had an acceptable long-term prognostic, but it was not possible to discern statistical differences between the three neoadjuvant subgroups. To further confirm the safety and efficacy of neoadjuvant PD-(L)1 inhibitors, more multicenter, randomized controlled trials and longer follow-up time are necessary.

## Data availability statement

The raw data supporting the conclusions of this article will be made available by the authors, without undue reservation.

## Author contributions

SH: Data curation, Software, Writing – original draft. YH: Data curation, Software, Writing – original draft. CL: Methodology, Project administration, Supervision, Writing – original draft. YL: Conceptualization, Data curation, Writing – original draft. MH: Investigation, Supervision, Writing – original draft. RL: Conceptualization, Funding acquisition, Resources, Writing – original draft. WL: Funding acquisition, Writing – review & editing.

## References

[1] Ploeg M Aben KKH Kiemeney LA . The present and future burden of urinary bladder cancer in the world. World J Urol (2009) 27(3):289–93. doi: 10.1007/s00345-009-0383-3 PMC2694323 19219610

[2] Guillaume L Guy L . Epidemiology of and risk factors for bladder cancer and for urothelial tumors. La Rev du praticien (2014) 64(10):1372–4, 8-80. 25668830

[3] Babjuk M Bohle A Burger M Capoun O Cohen D Comperat EM . Eau guidelines on non-muscle-invasive urothelial carcinoma of the bladder: update 2016. Eur Urol (2017) 71(3):447–61. doi: 10.1016/j.eururo.2016.05.041 27324428

[4] Cambier S Sylvester RJ Collette L Gontero P Brausi MA van Andel G . Eortc nomograms and risk groups for predicting recurrence, progression, and disease-specific and overall survival in non-muscle-invasive stage ta-T1 urothelial bladder cancer patients treated with 1-3 years of maintenance bacillus calmette-guerin. Eur Urol (2016) 69(1):60–9. doi: 10.1016/j.eururo.2015.06.045 26210894

[5] Dyrskjot L Zieger K Real FX Malats N Carrato A Hurst C . Gene expression signatures predict outcome in non-muscle invasive bladder carcinoma: A multicenter validation study. Clin Cancer Res (2007) 13(12):3545–51. doi: 10.1158/1078-0432.ccr-06-2940 17575217

[6] Fernandez-Gomez J Madero R Solsona E Unda M Martinez-Pineiro L Gonzalez M . Predicting nonmuscle invasive bladder cancer recurrence and progression in patients treated with bacillus calmette-guerin: the cueto scoring model. J Urol (2009) 182(5):2195–203. doi: 10.1016/j.juro.2009.07.016 19758621

[7] Edge SB Compton CC . The American joint committee on cancer: the 7th edition of the ajcc cancer staging manual and the future of tnm. Ann Surg Oncol (2010) 17(6):1471–4. doi: 10.1245/s10434-010-0985-4 20180029

[8] Kulkarni GS Black PC Sridhar SS Kapoor A Zlotta AR Shayegan B . Canadian urological association guideline: muscle-invasive bladder cancer. Cuaj-Canadian Urological Assoc J (2019) 13(8):230–8. doi: 10.5489/cuaj.5902 PMC6737737 30763236

[9] Witjes JA Bruins HM Cathomas R Comperat EM Cowan NC Gakis G . European association of urology guidelines on muscle-invasive and metastatic bladder cancer: summary of the 2020 guidelines. Eur Urol (2021) 79(1):82–104. doi: 10.1016/j.eururo.2020.03.055 32360052

[10] Lee Y Kim YS Hong B Cho YM Lee JL . Comparison of clinical outcomes in patients with localized or locally advanced urothelial carcinoma treated with neoadjuvant chemotherapy involving gemcitabine-cisplatin and high dose-intensity mvac. J Cancer Res Clin Oncol (2021) 147(11):3421–9. doi: 10.1007/s00432-021-03582-x PMC11801947 33715088

[11] Ravi P Pond GR Diamantopoulos LN Su C Alva A Jain RK . Optimal pathological response after neoadjuvant chemotherapy for muscle-invasive bladder cancer: results from a global, multicentre collaboration. Bju Int (2021) 128(5):607–14. doi: 10.1111/bju.15434 33909949

[12] Funt SA Rosenberg JE . Systemic, perioperative management of muscle-invasive bladder cancer and future horizons. Nat Rev Clin Oncol (2017) 14(4):221–34. doi: 10.1038/nrclinonc.2016.188 PMC6054138 27874062

[13] Pfister C Gravis G Fléchon A Soulié M Guy L Laguerre B . Randomized phase iii trial of dose-dense methotrexate, vinblastine, doxorubicin, and cisplatin, or gemcitabine and cisplatin as perioperative chemotherapy for patients with muscle-invasive bladder cancer. Analysis of the getug/afu V05 vesper trial secondary endpoints: chemotherapy toxicity and pathological responses. Eur Urol (2021) 79(2):214–21. doi: 10.1016/j.eururo.2020.08.024 32868138

[14] Galsky MD Hahn NM Rosenberg JE Sonpavde G Oh WK Dreicer R . Defining "Cisplatin ineligible" Patients with metastatic bladder cancer. J Clin Oncol (2011) 29(7):238. doi: 10.1200/jco.2011.29.7_suppl.238 21555688

[15] Choueiri TK Powles T Burotto M Escudier B Bourlon MT Zurawski B . Nivolumab plus cabozantinib versus sunitinib for advanced renal-cell carcinoma. New Engl J Med (2021) 384(9):829–41. doi: 10.1056/NEJMoa2026982 PMC8436591 33657295

[16] Hellmann MD Paz-Ares L Caro RB Zurawski B Kim SW Costa EC . Nivolumab plus ipilimumab in advanced non-small-cell lung cancer. New Engl J Med (2019) 381(21):2020–31. doi: 10.1056/NEJMoa1910231 31562796

[17] Hodi FS Chiarion-Sileni V Gonzalez R Grob JJ Rutkowski P Cowey CL . Nivolumab plus ipilimumab or nivolumab alone versus ipilimumab alone in advanced melanoma (Checkmate 067): 4-year outcomes of a multicentre, randomised, phase 3 trial. Lancet Oncol (2018) 19(11):1480–92. doi: 10.1016/s1470-2045(18)30700-9 30361170

[18] Motzer RJ Tannir NM McDermott DF Frontera OA Melichar B Choueiri TK . Nivolumab plus ipilimumab versus sunitinib in advanced renal-cell carcinoma. New Engl J Med (2018) 378(14):1277–90. doi: 10.1056/NEJMoa1712126 PMC5972549 29562145

[19] Sonpavde G . Pd-1 and pd-L1 inhibitors as salvage therapy for urothelial carcinoma. N Engl J Med (2017) 376(11):1073–4. doi: 10.1056/NEJMe1701182 28212061

[20] Bellmunt J Powles T Vogelzang NJ . A review on the evolution of pd-1/pd-L1 immunotherapy for bladder cancer: the future is now. Cancer Treat Rev (2017) 54:58–67. doi: 10.1016/j.ctrv.2017.01.007 28214651

[21] Galsky MD Arija JÁA Bamias A Davis ID De Santis M Kikuchi E . Atezolizumab with or without chemotherapy in metastatic urothelial cancer (Imvigor130): A multicentre, randomised, placebo-controlled phase 3 trial. Lancet (London England) (2020) 395(10236):1547–57. doi: 10.1016/s0140-6736(20)30230-0 32416780

[22] Powles T Csoszi T Ozguroglu M Matsubara N Geczi L Cheng SYS . Pembrolizumab alone or combined with chemotherapy versus chemotherapy as first-line therapy for advanced urothelial carcinoma (Keynote-361): A randomised, open-label, phase 3 trial. Lancet Oncol (2021) 22(7):931–45. doi: 10.1016/s1470-2045(21)00152-2 34051178

[23] Powles T Kockx M Rodriguez-Vida A Duran I Crabb SJ van der Heijden MS . Clinical efficacy and biomarker analysis of neoadjuvant atezolizumab in operable urothelial carcinoma in the abacus trial. Nat Med (2019) 25(11):1706–14. doi: 10.1038/s41591-019-0628-7 31686036

[24] Necchi A Anichini A Raggi D Briganti A Massa S Lucianò R . Pembrolizumab as neoadjuvant therapy before radical cystectomy in patients with muscle-invasive urothelial bladder carcinoma (Pure-01): an open-label, single-arm, phase ii study. J Clin Oncol (2018) 36(34):3353–60. doi: 10.1200/jco.18.01148 30343614

[25] Hahn NM Necchi A Loriot Y Powles T Plimack ER Sonpavde G . Role of checkpoint inhibition in localized bladder cancer. Eur Urol Oncol (2018) 1(3):190–8. doi: 10.1016/j.euo.2018.05.002 31102620

[26] Basile G Bandini M Gibb EA Ross JS Raggi D Marandino L . Neoadjuvant pembrolizumab and radical cystectomy in patients with muscle-invasive urothelial bladder cancer: 3-year median follow-up update of pure-01 trial. Clin Cancer Res (2022) 28(23):5107–14. doi: 10.1158/1078-0432.ccr-22-2158 36190522

[27] Necchi A Raggi D Gallina A Madison R Colecchia M Luciano R . Updated results of pure-01 with preliminary activity of neoadjuvant pembrolizumab in patients with muscle-invasive bladder carcinoma with variant histologies. Eur Urol (2020) 77(4):439–46. doi: 10.1016/j.eururo.2019.10.026 31708296

[28] Kim H Jeong BC Hong J Kwon GY Kim CK Park W . Neoadjuvant nivolumab plus gemcitabine/cisplatin chemotherapy in muscle-invasive urothelial carcinoma of the bladder. Cancer Res Treat (2023) 55(2):636–42. doi: 10.4143/crt.2022.343 PMC10101782 36228654

[29] van Dijk N Gil-Jimenez A Silina K Hendricksen K Smit LA de Feijter JM . Preoperative ipilimumab plus nivolumab in locoregionally advanced urothelial cancer: the nabucco trial. Nat Med (2020) 26(12):1839–44. doi: 10.1038/s41591-020-1085-z 33046870

[30] Bañares R Albillos A Rincón D Alonso S González M Ruiz-del-Arbol L . Endoscopic treatment versus endoscopic plus pharmacologic treatment for acute variceal bleeding: A meta-analysis. Hepatol (Baltimore Md) (2002) 35(3):609–15. doi: 10.1053/jhep.2002.31354 11870374

[31] Slim K Nini E Forestier D Kwiatkowski F Panis Y Chipponi J . Methodological index for non-randomized studies (Minors): development and validation of a new instrument. ANZ J Surg (2003) 73(9):712–6. doi: 10.1046/j.1445-2197.2003.02748.x 12956787

[32] Liu N Zhou Y Lee JJ . Ipdfromkm: reconstruct individual patient data from published kaplan-meier survival curves. BMC Med Res Method (2021) 21(1):111. doi: 10.1186/s12874-021-01308-8 PMC8168323 34074267

[33] Page MJ McKenzie JE Bossuyt PM Boutron I Hoffmann TC Mulrow CD . The PRISMA 2020 statement: An updated guideline for reporting systematic reviews. J Clin Epidemiol (2021) 134:178-89. doi: 10.1016/j.jclinepi.2021.03.001 33789819

[34] Goubet AG Silva CAC De Melo LL Gazzano M Lebacle C Thibault C . Bacteria-specific cxcl13-producing follicular helper T cells are putative prognostic markers to neoadjuvant pd-1 blockade in muscle-invasive urothelial carcinoma. J Clin Oncol (2022) 40(6):535. doi: 10.1200/JCO.2022.40.6_suppl.535

[35] Szabados B Rodriguez-Vida A Durán I Crabb SJ van der Heijden MS Pous AF . Toxicity and surgical complication rates of neoadjuvant atezolizumab in patients with muscle-invasive bladder cancer undergoing radical cystectomy: updated safety results from the abacus trial. Eur Urol Oncol (2021) 4(3):456–63. doi: 10.1016/j.euo.2020.11.010 33612455

[36] Koshkin VS Natesan D Zhang L Oh DY Porten SP Meng M . Phase ii trial of escalating doses of neoadjuvant atezolizumab for patients with non-metastatic urothelial carcinoma ineligible for cisplatin-based neoadjuvant chemotherapy. J Clin Oncol (2021) 39(6_suppl):442–. doi: 10.1200/JCO.2021.39.6_suppl.442

[37] Guercio BJ Pietzak EJ Brown S Chen J-F Peters V Regazzi AM . Neoadjuvant nivolumab (N) +/- ipilimumab (I) in cisplatin-ineligible patients (Pts) with muscle-invasive bladder cancer (Mibc). J Clin Oncol (2022) 40(6_suppl):498–. doi: 10.1200/JCO.2022.40.6_suppl.498

[38] Grivas P Yin J Koshkin VS Cole S Jain RK Dreicer R . Pre0807: A phase ib feasibility trial of neoadjuvant nivolumab (N) without or with lirilumab (L) in cisplatin-ineligible patients (Pts) with muscle-invasive bladder cancer (Mibc). J Clin Oncol (2021) 39(15):4518. doi: 10.1200/JCO.2021.39.15_suppl.4518

[39] Wei XX McGregor BA Lee RJ Gao X Kilbridge KL Preston MA . Durvalumab as neoadjuvant therapy for muscle-invasive bladder cancer: preliminary results from the bladder cancer signal seeking trial (Blasst)-2. J Clin Oncol (2020) 38(6):507. doi: 10.1200/JCO.2020.38.6_suppl.507

[40] Martinez Chanza N Carnot A Barthélémy P Casert V Staudacher L Van Den Brande J . Avelumab as the basis of neoadjuvant regimen in platinum-eligible and -ineligible patients with nonmetastatic muscle-invasive bladder cancer: aura (Oncodistinct-004) trial. J Clin Oncol (2022) 40(16_suppl):4517–. doi: 10.1200/JCO.2022.40.16_suppl.4517

[41] Van Dorp J Suelmann BBM Mehra N Van Montfoort M Van Dijk N Hendricksen K . Lba31 high- vs low-dose pre-operative ipilimumab and nivolumab in locoregionally advanced urothelial cancer (Nabucco cohort 2). Ann Oncol (2021) 32:S1305–S6. doi: 10.1016/j.annonc.2021.08.2107

[42] Grande E Guerrero F Puente J Galante I Duran I Dominguez M . Dutreneo trial: A randomized phase ii trial of durvalumab and tremelimumab versus chemotherapy as a neoadjuvant approach to muscle-invasive urothelial bladder cancer (Mibc) patients (Pts) prospectively selected by an interferon (Inf)-gamma immune signature. J Clin Oncol (2020) 38(15_suppl):5012. doi: 10.1200/JCO.2020.38.15_suppl.5012

[43] Gao J Navai N Alhalabi O Siefker-Radtke A Campbell MT Tidwell RS . Neoadjuvant pd-L1 plus ctla-4 blockade in patients with cisplatin-ineligible operable high-risk urothelial carcinoma. Nat Med (2020) 26(12):1845–51. doi: 10.1038/s41591-020-1086-y PMC9768836 33046869

[44] Xing N Han S Jiang J Xu W Shi B Ping H . 703p camrelizumab in combination with gemcitabine plus cisplatin as neoadjuvant therapy for muscle-invasive bladder cancer. Ann Oncol (2021) 32:S714. doi: 10.1016/j.annonc.2021.08.099

[45] Rose TL Harrison MR Deal AM Ramalingam S Whang YE Brower B . Phase ii study of gemcitabine and split-dose cisplatin plus pembrolizumab as neoadjuvant therapy before radical cystectomy in patients with muscle-invasive bladder cancer. J Clin Oncol Off J Am Soc Clin Oncol (2021) 39(28):3140–8. doi: 10.1200/jco.21.01003 PMC8478388 34428076

[46] Lin T Li K Fan J Wang S Yu D Xu T . Interim results from a multicenter clinical study of tislelizumab combined with gemcitabine and cisplatin as neoadjuvant therapy for patients with ct2-T4an0m0 mibc. J Clin Oncol (2022) 40(16_suppl):4580. doi: 10.1200/JCO.2022.40.16_suppl.4580

[47] Kaimakliotis H Albany C Hoffman-Censits J Trabulsi E Kelly WK Picus J . Pd52-03 a multicenter phase 1b/2 study of neoadjuvant pembrolizumab and cisplatin chemotherapy for muscle invasive urothelial cancer. J Urol (2019) 201(Supplement 4):e924–e5. doi: 10.1097/01.JU.0000556959.45525.89

[48] Gupta S Gibb E Sonpavde GP Gupta S Maughan BL Agarwal N . Biomarker analysis and updated clinical follow-up from blasst-1 (Bladder cancer signal seeking trial) of nivolumab, gemcitabine, and cisplatin in patients with muscle-invasive bladder cancer (Mibc) undergoing cystectomy. J Clin Oncol (2022) 40(6_suppl):528. doi: 10.1200/JCO.2022.40.6_suppl.528

[49] Funt SA Lattanzi M Whiting K Al-Ahmadie H Quinlan C Teo MY . Neoadjuvant atezolizumab with gemcitabine and cisplatin in patients with muscle-invasive bladder cancer: A multicenter, single-arm, phase ii trial. J Clin Oncol (2022) 40(12):1312–22. doi: 10.1200/jco.21.01485 PMC9797229 35089812

[50] Cathomas R Petrausch U Hayoz S Schneider M Schardt JA Seiler R . Perioperative chemoimmunotherapy with durvalumab (Durva) in combination with cisplatin/gemcitabine (Cis/gem) for operable muscle-invasive urothelial carcinoma (Miuc): preplanned interim analysis of a single-arm phase ii trial (Sakk 06/17). J Clin Oncol (2020) 38(6_suppl):499. doi: 10.1200/JCO.2020.38.6_suppl.499

[51] Thibault C Elaidi R Vano YA Rouabah M Braychenko E Helali I . Open-label phase ii to evaluate the efficacy of neoadjuvant dose-dense mvac in combination with durvalumab and tremelimumab in muscle-invasive urothelial carcinoma: nemio. Bull du Cancer (2020) 107(5s):eS8–eS15. doi: 10.1016/s0007-4551(20)30281-2 32620213

[52] Kaimakliotis HZ Adra N Kelly WK Trabulsi EJ Lauer RC Picus J . Phase ii neoadjuvant (N-) gemcitabine (G) and pembrolizumab (P) for locally advanced urothelial cancer (Lauc): interim results from the cisplatin (C)-ineligible cohort of gu14-188. J Clin Oncol (2020) 38(15_suppl):5019. doi: 10.1200/JCO.2020.38.15_suppl.5019

[53] Han S Ji Z Jiang J Fan X Ma Q Hu L . Neoadjuvant therapy with camrelizumab plus gemcitabine and cisplatin for patients with muscle-invasive bladder cancer: A multi-center, single-arm, phase 2 study. Cancer Med (2023) 12(11):12106–17. doi: 10.1002/cam4.5900 PMC10278497 37021811

[54] Szabados B Kockx M Assaf ZJ van Dam PJ Rodriguez-Vida A Duran I . Final results of neoadjuvant atezolizumab in cisplatin-ineligible patients with muscle-invasive urothelial cancer of the bladder. Eur Urol (2022) 82(2):212–22. doi: 10.1016/j.eururo.2022.04.013 35577646

[55] Takahashi T . Comments on "Survival after neoadjuvant/induction combination immunotherapy versus combination platinum-based chemotherapy for locally advanced (Stage iii) urothelial cancer". Int J Cancer (2022) 151(10):1847–8. doi: 10.1002/ijc.34206 35815951

[56] Forde PM Chaft JE Smith KN Anagnostou V Cottrell TR Hellmann MD . Neoadjuvant pd-1 blockade in resectable lung cancer. New Engl J Med (2018) 378(21):1976–86. doi: 10.1056/NEJMoa1716078 PMC6223617 29658848

[57] Rozeman EA Blank CU Van Akkooi ACJ Kvistborg P Fanchi L Van Thienen JV . Neoadjuvant ipilimumab + Nivolumab (Ipi+Nivo) in palpable stage iii melanoma: updated data from the opacin trial and first immunological analyses. J Clin Oncol (2017) 35(15):9586. doi: 10.1200/JCO.2017.35.15_suppl.9586

[58] Forde PM Spicer J Lu S Provencio M Mitsudomi T Awad MM . Neoadjuvant nivolumab plus chemotherapy in resectable lung cancer. New Engl J Med (2022) 386(21):1973–85. doi: 10.1056/NEJMoa2202170 PMC9844511 35403841

[59] Schmid P Park YH Munoz-Couselo E Kim SB Sohn J Im SA . Pembrolizumab (Pembro) plus chemotherapy (Chemo) as neoadjuvant treatment for triple negative breast cancer (Tnbc): preliminary results from keynote-173. J Clin Oncol (2017) 35:556. doi: 10.1200/JCO.2017.35.15_suppl.556

[60] Broderick SR . Adjuvant and neoadjuvant immunotherapy in non - small cell lung cancer. Thorac Surg Clinics (2020) 30(2):215. doi: 10.1016/j.thorsurg.2020.01.001 32327180

[61] Owen D Chaft JE . Immunotherapy in surgically resectable non-small cell lung cancer. J Thorac Dis (2018) 10(Suppl 3):S404–s11. doi: 10.21037/jtd.2017.12.93 PMC5861266 29593886

[62] Liu J Blake SJ Yong MCR Harjunpaa H Ngiow SF Takeda K . Improved efficacy of neoadjuvant compared to adjuvant immunotherapy to eradicate metastatic disease. Cancer Discovery (2016) 6(12):1382–99. doi: 10.1158/2159-8290.cd-16-0577 27663893

[63] Powles T van der Heijden MS Castellano D Galsky MD Loriot Y Petrylak DP . Durvalumab alone and durvalumab plus tremelimumab versus chemotherapy in previously untreated patients with unresectable, locally advanced or metastatic urothelial carcinoma (Danube): A randomised, open-label, multicentre, phase 3 trial. Lancet Oncol (2020) 21(12):1574–88. doi: 10.1016/s1470-2045(20)30541-6 32971005

[64] Iyer G Tully CM Zabor EC Bochner BH Dalbagni G Herr HW . Neoadjuvant gemcitabine-cisplatin plus radical cystectomy-pelvic lymph node dissection for muscle-invasive bladder cancer: A 12-year experience. Clin genitourinary Cancer (2020) 18(5):387–94. doi: 10.1016/j.clgc.2020.02.014 PMC8375301 32273235

[65] Rosenberg JE Hoffman-Censits J Powles T van der Heijden MS Balar AV Necchi A . Atezolizumab in patients with locally advanced and metastatic urothelial carcinoma who have progressed following treatment with platinum-based chemotherapy: A single-arm, multicentre, phase 2 trial. Lancet (London England) (2016) 387(10031):1909–20. doi: 10.1016/s0140-6736(16)00561-4 PMC5480242 26952546

[66] Sharma P Retz M Siefker-Radtke A Baron A Necchi A Bedke J . Nivolumab in metastatic urothelial carcinoma after platinum therapy (Checkmate 275): A multicentre, single-arm, phase 2 trial. Lancet Oncol (2017) 18(3):312–22. doi: 10.1016/s1470-2045(17)30065-7 28131785

[67] Massard C Gordon MS Sharma S Rafii S Wainberg ZA Luke J . Safety and efficacy of durvalumab (Medi4736), an anti-programmed cell death ligand-1 immune checkpoint inhibitor, in patients with advanced urothelial bladder cancer. J Clin Oncol (2016) 34(26):3119–25. doi: 10.1200/jco.2016.67.9761 PMC5569690 27269937

[68] Powles T Park SH Voog E Caserta C Valderrama BP Gurney H . Maintenance avelumab + Best supportive care (Bsc) versus bsc alone after platinum-based first-line (1l) chemotherapy in advanced urothelial carcinoma (Uc): javelin bladder 100 phase iii interim analysis. J Clin Oncol (2020) 38(18_suppl):LBA1–LBA. doi: 10.1200/JCO.2020.38.18_suppl.LBA1

[69] Zargar H Shah JB van Rhijn BW Daneshmand S Bivalacqua TJ Spiess PE . Neoadjuvant dose dense mvac versus gemcitabine and cisplatin in patients with ct3-4an0m0 bladder cancer treated with radical cystectomy. J Urol (2018) 199(6):1453–9. doi: 10.1016/j.juro.2017.12.062 29329894

[70] Peyton CC Tang D Reich RR Azizi M Chipollini J Pow-Sang JM . Downstaging and survival outcomes associated with neoadjuvant chemotherapy regimens among patients treated with cystectomy for muscle-invasive bladder cancer. JAMA Oncol (2018) 4(11):1535–42. doi: 10.1001/jamaoncol.2018.3542 PMC6248089 30178038

[71] Van Der Heijden MS Gupta S Galsky MD Derleth CL Lee S Kataria RS . Study ev-302: A two-arm, open-label, randomized controlled phase 3 study of enfortumab vedotin in combination with pembrolizumab versus chemotherapy in previously untreated advanced urothelial carcinoma (Auc) (Trial in progress). J Clin Oncol (2022) 40(6_suppl):TPS589–TPS. doi: 10.1200/JCO.2022.40.6_suppl.TPS589

[72] Hoimes CJ Bedke J Loriot Y Nishiyama H Fang X Kataria RS . Keynote-B15/ev-304: randomized phase 3 study of perioperative enfortumab vedotin plus pembrolizumab versus chemotherapy in cisplatin-eligible patients with muscle-invasive bladder cancer (Mibc). J Clin Oncol (2021) 39(15_suppl):TPS4587–TPS. doi: 10.1200/JCO.2021.39.15_suppl.TPS4587

[73] Eriksson V Holmlund J Wiberg E Johansson M Huge Y Alamdari F . Adverse events during neoadjuvant chemotherapy for muscle invasive bladder cancer-a Swedish retrospective multicentre study of a clinical database. Trans Androl Urol (2022) 11(8):1105–15. doi: 10.21037/tau-22-78 PMC9459545 36092838

[74] Frenel JS Le Tourneau C O'Neil B Ott PA Piha-Paul SA Gomez-Roca C . Safety and efficacy of pembrolizumab in advanced, programmed death ligand 1-positive cervical cancer: results from the phase ib keynote-028 trial. J Clin Oncol (2017) 35(36):4035–41. doi: 10.1200/jco.2017.74.5471 29095678

[75] Necchi A Joseph RW Loriot Y Hoffman-Censits J Perez-Gracia JL Petrylak DP . Atezolizumab in platinum-treated locally advanced or metastatic urothelial carcinoma: post-progression outcomes from the phase ii imvigor210 study. Ann Oncol (2017) 28(12):3044–50. doi: 10.1093/annonc/mdx518 PMC5834063 28950298

[76] Grunewald CM Niegisch G Albers P . Using circulating tumor DNA to guide adjuvant therapy in bladder cancer: imvigor010 and imvigor011. Eur Urol Focus (2022) 8(3):646–7. doi: 10.1016/j.euf.2022.04.001 35450799

[77] Bellmunt J Hussain M Gschwend JE Albers P Oudard S Castellano D . Adjuvant atezolizumab versus observation in muscle-invasive urothelial carcinoma (Imvigor010): A multicentre, open-label, randomised, phase 3 trial. Lancet Oncol (2021) 22(4):525–37. doi: 10.1016/s1470-2045(21)00004-8 PMC8495594 33721560

