# Supplementary material for: Efficacy and safety of neoadjuvant PD-1 inhibitors or PD-L1 inhibitors for muscle invasive bladder cancer: a systematic review and meta-analysis
Source: Front Immunol. 2024 Jan 9;14:1332213. doi: 10.3389/fimmu.2023.1332213 (PMC10803485; doi:10.3389/fimmu.2023.1332213)
Supplement: Supplementary file 1 [file Table_1.docx]

1.干预：PD-1 inhibitor （具体药物：Pembrolizumab、Nivolumab、Toripalimab、Tislelizumab、Camrelizumab、GLS-010、Cemiplimab、Sintilimab、Zimberelimab、Prolgolimab、Dostarlimab）

或PD-L1 inhibitor（具体药物：Atezolizumab、Durvalumab、Avelumab）

2.疾病：Muscle-invasive Bladder Carcinoma

3.RCT、前瞻性研究、回顾性研究

4.Neoadjuvant therapy（新辅助治疗）

**PD-1 inhibitor**

PD-1 Inhibitors

PD 1 Inhibitors

PD-1 Inhibitor

Inhibitor, PD-1

PD 1 Inhibitor

Programmed Cell Death Protein 1 Inhibitor

Programmed Cell Death Protein 1 Inhibitors

Pembrolizumab

SCH-900475

Lambrolizumab

MK-3475

Keytruda

Nivolumab

Opdivo

ONO-4538

ONO 4538

ONO4538

MDX-1106

MDX 1106

MDX1106

BMS-936558

BMS 936558

BMS936558

Toripalimab

Tislelizumab

BGB-A317

Camrelizumab

carrelizumab

SHR-1210

SHR 1210

GLS-010

Cemiplimab

REGN2810

Sintilimab

IBI308

IBI-308

Zimberelimab

GLS-010

Prolgolimab

Dostarlimab

Jemperli

dostarlimab-gxly

TSR-042

GSK4057190

**PD-L1 inhibitor**

PD-L1 Inhibitors

PD L1 Inhibitors

PD-L1 Inhibitor

PD L1 Inhibitor

Programmed Death-Ligand 1 Inhibitors

Programmed Death Ligand 1 Inhibitors

PD-1-PD-L1 Blockade

Blockade, PD-1-PD-L1

PD 1 PD L1 Blockade

Atezolizumab

anti-PDL1

immunoglobulin G1, anti-(human CD antigen CD274) (human monoclonal MDPL3280a heavy chain), disulfide with human monoclonal MDPL3280a kappa-chain, dimer

MPDL3280A

MPDL-3280A

Tecentriq

RG7446

RG-7446

Durvalumab

MEDI4736

MEDI-4736

Imfinzi

Avelumab

MSB-0010682

MSB0010682

bavencio

MSB0010718C

MSB-0010718C

膀胱癌

Urinary Bladder Neoplasms

Neoplasm, Urinary Bladder

Urinary Bladder Neoplasm

Bladder Tumors

Bladder Tumor

Tumor, Bladder

Tumors, Bladder

Neoplasms, Bladder

Bladder Neoplasms

Bladder Neoplasm

Neoplasm, Bladder

Urinary Bladder Cancer

Cancer, Urinary Bladder

Malignant Tumor of Urinary Bladder

Cancer of the Bladder

Bladder Cancer

Bladder Cancers

Cancer, Bladder

Cancer of Bladder

RCT

randomized controlled trial

controlled clinical trial

Randomized

placebo

clinical trials as topic

randomly

Trial

前瞻性研究

Prospective Studies

Prospective Study

Studies, Prospective

Study, Prospective

回顾性研究

Retrospective Studies

Studies, Retrospective

Study, Retrospective

Retrospective Study

新辅助治疗

Neoadjuvant therapy

Neoadjuvant Therapies

Therapy, Neoadjuvant

Neoadjuvant Treatment

Neoadjuvant Treatments

Treatment, Neoadjuvant

Neoadjuvant Chemoradiotherapy

Chemoradiotherapy, Neoadjuvant

Neoadjuvant Chemoradiotherapies

Neoadjuvant Chemoradiation Therapy

Chemoradiation Therapy, Neoadjuvant

Neoadjuvant Chemoradiation Therapies

Therapy, Neoadjuvant Chemoradiation

Neoadjuvant Chemoradiation Treatment

Chemoradiation Treatment, Neoadjuvant

Neoadjuvant Chemoradiation Treatments

Treatment, Neoadjuvant Chemoradiation

Neoadjuvant Chemoradiation

Chemoradiation, Neoadjuvant

Neoadjuvant Chemoradiations

Neoadjuvant Radiotherapy

Neoadjuvant Radiotherapies

Radiotherapy, Neoadjuvant

Neoadjuvant Radiation Treatment

Neoadjuvant Radiation Treatments

Radiation Treatment, Neoadjuvant

Treatment, Neoadjuvant Radiation

Neoadjuvant Radiation Therapy

Neoadjuvant Radiation Therapies

Radiation Therapy, Neoadjuvant

Therapy, Neoadjuvant Radiation

Neoadjuvant Radiation

Neoadjuvant Radiations

Radiation, Neoadjuvant

Neoadjuvant Chemotherapy

Chemotherapy, Neoadjuvant

Neoadjuvant Chemotherapies

Neoadjuvant Chemotherapy Treatment

Chemotherapy Treatment, Neoadjuvant

Neoadjuvant Chemotherapy Treatments

Treatment, Neoadjuvant Chemotherapy

Neoadjuvant Systemic Therapy

Neoadjuvant Systemic Therapies

Systemic Therapy, Neoadjuvant

Therapy, Neoadjuvant Systemic

Neoadjuvant Systemic Treatment

Neoadjuvant Systemic Treatments

Systemic Treatment, Neoadjuvant

Treatment, Neoadjuvant Systemic

| PubMed | | |
| --- | --- | --- |
| No. | Query | Results |
| 1 | (((((((((((((((((((((((((((((((((((((((((((((((((((((((((((((((((((((((PD-1 inhibitor[Title/Abstract]) OR (Pembrolizumab[Title/Abstract])) OR (SCH-900475[Title/Abstract])) OR (Lambrolizumab[Title/Abstract])) OR (MK-3475[Title/Abstract])) OR (Keytruda[Title/Abstract])) OR (Nivolumab[Title/Abstract])) OR (Opdivo[Title/Abstract])) OR (ONO-4538[Title/Abstract])) OR (ONO 4538[Title/Abstract])) OR (ONO4538[Title/Abstract])) OR (MDX-1106[Title/Abstract])) OR (MDX 1106[Title/Abstract])) OR (MDX1106[Title/Abstract])) OR (BMS-936558[Title/Abstract])) OR (BMS 936558[Title/Abstract])) OR (BMS936558[Title/Abstract])) OR (Toripalimab[Title/Abstract])) OR (Tislelizumab[Title/Abstract])) OR (BGB-A317[Title/Abstract])) OR (Camrelizumab[Title/Abstract])) OR (carrelizumab[Title/Abstract])) OR (SHR-1210[Title/Abstract])) OR (SHR 1210[Title/Abstract])) OR (GLS-010[Title/Abstract])) OR (Cemiplimab[Title/Abstract])) OR (REGN2810[Title/Abstract])) OR (Sintilimab[Title/Abstract])) OR (IBI308[Title/Abstract])) OR (IBI-308[Title/Abstract])) OR (Zimberelimab[Title/Abstract])) OR (Prolgolimab[Title/Abstract])) OR (Dostarlimab[Title/Abstract])) OR (Jemperli[Title/Abstract])) OR (dostarlimab-gxly[Title/Abstract])) OR (TSR-042[Title/Abstract])) OR (GSK4057190[Title/Abstract])) OR (PD-L1 inhibitor[Title/Abstract])) OR (Atezolizumab[Title/Abstract])) OR (anti-PDL1[Title/Abstract])) OR (immunoglobulin G1, anti-(human CD antigen CD274) (human monoclonal MDPL3280a heavy chain), disulfide with human monoclonal MDPL3280a kappa-chain, dimer[Title/Abstract])) OR (MPDL3280A[Title/Abstract])) OR (MPDL-3280A[Title/Abstract])) OR (Tecentriq[Title/Abstract])) OR (RG7446[Title/Abstract])) OR (RG-7446[Title/Abstract])) OR (Durvalumab[Title/Abstract])) OR (MEDI4736[Title/Abstract])) OR (MEDI-4736[Title/Abstract])) OR (Imfinzi[Title/Abstract])) OR (Avelumab[Title/Abstract])) OR (MSB-0010682[Title/Abstract])) OR (MSB0010682[Title/Abstract])) OR (bavencio[Title/Abstract])) OR (MSB0010718C[Title/Abstract])) OR (MSB-0010718C[Title/Abstract])) OR (PD-1 Inhibitors[Title/Abstract])) OR (PD 1 Inhibitors[Title/Abstract])) OR (PD-1 Inhibitor[Title/Abstract])) OR (Inhibitor, PD-1[Title/Abstract])) OR (PD 1 Inhibitor[Title/Abstract])) OR (Programmed Cell Death Protein 1 Inhibitor[Title/Abstract])) OR (Programmed Cell Death Protein 1 Inhibitors[Title/Abstract])) OR (PD-L1 Inhibitors[Title/Abstract])) OR (PD L1 Inhibitors[Title/Abstract])) OR (PD-L1 Inhibitor[Title/Abstract])) OR (PD L1 Inhibitor[Title/Abstract])) OR (Programmed Death-Ligand 1 Inhibitors[Title/Abstract])) OR (Programmed Death Ligand 1 Inhibitors[Title/Abstract])) OR (PD-1-PD-L1 Blockade[Title/Abstract])) OR (Blockade, PD-1-PD-L1[Title/Abstract])) OR (PD 1 PD L1 Blockade[Title/Abstract]) | 21,408 results |
| 2 | ((((((((((((((((((Urinary Bladder Neoplasms[Title/Abstract]) OR (Neoplasm, Urinary Bladder[Title/Abstract])) OR (Urinary Bladder Neoplasm[Title/Abstract])) OR (Bladder Tumors[Title/Abstract])) OR (Bladder Tumor[Title/Abstract])) OR (Tumor, Bladder[Title/Abstract])) OR (Tumors, Bladder[Title/Abstract])) OR (Neoplasms, Bladder[Title/Abstract])) OR (Bladder Neoplasms[Title/Abstract])) OR (Bladder Neoplasm[Title/Abstract])) OR (Neoplasm, Bladder[Title/Abstract])) OR (Urinary Bladder Cancer[Title/Abstract])) OR (Cancer, Urinary Bladder[Title/Abstract])) OR (Malignant Tumor of Urinary Bladder[Title/Abstract])) OR (Cancer of the Bladder[Title/Abstract])) OR (Bladder Cancer[Title/Abstract])) OR (Bladder Cancers[Title/Abstract])) OR (Cancer, Bladder[Title/Abstract])) OR (Cancer of Bladder[Title/Abstract]) | 52,023 results |
| 3 | (((randomized controlled trial [pt] OR controlled clinical trial [pt] OR randomized [tiab] OR placebo [tiab] OR clinical trials as topic [mesh: noexp] OR randomly [tiab] OR trial [ti]) NOT (animals [mh] NOT humans [mh])) OR (Prospective[Title/Abstract])) OR (Retrospective[Title/Abstract]) | 2,745,392 results |
| 4 | Neoadjuvant | 55,952 results |
| 5 | 1 AND 2 AND 4 | 118 results |
| 6 | 1 AND 2 AND 3 AND 4 | 49results |

| Web of science | | |
| --- | --- | --- |
| No. | Query | Results |
| 1 | (((((((((((((((((((((((((((((((((((((((((((((((((((((((((((((((((((((((TS=(PD-1 inhibitor )) OR TS=(PD-1 Inhibitors)) OR TS=(PD 1 Inhibitors)) OR TS=(PD-1 Inhibitor)) OR TS=(Inhibitor, PD-1)) OR TS=(PD 1 Inhibitor)) OR TS=(Programmed Cell Death Protein 1 Inhibitor)) OR TS=(Programmed Cell Death Protein 1 Inhibitors)) OR TS=( Pembrolizumab)) OR TS=(SCH-900475)) OR TS=(Lambrolizumab)) OR TS=(MK-3475)) OR TS=(Keytruda)) OR TS=(Nivolumab)) OR TS=(Opdivo)) OR TS=(ONO-4538)) OR TS=(ONO 4538)) OR TS=(ONO4538)) OR TS=(MDX-1106)) OR TS=(MDX 1106)) OR TS=(MDX1106)) OR TS=(BMS-936558)) OR TS=(BMS 936558)) OR TS=(BMS936558)) OR TS=(Toripalimab)) OR TS=(Tislelizumab)) OR TS=(BGB-A317)) OR TS=(Camrelizumab)) OR TS=(carrelizumab)) OR TS=(SHR-1210)) OR TS=(SHR 1210)) OR TS=(GLS-010)) OR TS=(Cemiplimab)) OR TS=(REGN2810)) OR TS=(Sintilimab)) OR TS=(IBI308)) OR TS=(IBI-308)) OR TS=( Zimberelimab)) OR TS=(Prolgolimab)) OR TS=( Dostarlimab)) OR TS=(Jemperli)) OR TS=(dostarlimab-gxly)) OR TS=(TSR-042)) OR TS=(GSK4057190)) OR TS=(PD-L1 inhibitor)) OR TS=(PD-L1 Inhibitors)) OR TS=(PD L1 Inhibitors)) OR TS=(PD-L1 Inhibitor)) OR TS=(PD L1 Inhibitor)) OR TS=(Programmed Death-Ligand 1 Inhibitors)) OR TS=(Programmed Death Ligand 1 Inhibitors)) OR TS=(PD-1-PD-L1 Blockade)) OR TS=(Blockade, PD-1-PD-L1)) OR TS=(PD 1 PD L1 Blockade)) OR TS=(Atezolizumab)) OR TS=(anti-PDL1)) OR TS=(immunoglobulin G1, anti-(human CD antigen CD274) (human monoclonal MDPL3280a heavy chain), disulfide with human monoclonal MDPL3280a kappa-chain, dimer)) OR TS=(MPDL3280A)) OR TS=(MPDL-3280A)) OR TS=(Tecentriq)) OR TS=(RG7446)) OR TS=(RG-7446)) OR TS=(Durvalumab)) OR TS=(MEDI4736)) OR TS=(MEDI-4736)) OR TS=(Imfinzi)) OR TS=(Avelumab)) OR TS=(MSB-0010682)) OR TS=(MSB0010682)) OR TS=(bavencio)) OR TS=(MSB0010718C)) OR TS=(MSB-0010718C) | 87,059 results |
| 2 | ((((((((((((((((((TS=(Urinary Bladder Neoplasms)) OR TS=(Neoplasm, Urinary Bladder)) OR TS=(Urinary Bladder Neoplasm)) OR TS=(Bladder Tumors)) OR TS=(Bladder Tumor)) OR TS=(Tumor, Bladder)) OR TS=(Tumors, Bladder)) OR TS=(Neoplasms, Bladder)) OR TS=(Bladder Neoplasms)) OR TS=(Bladder Neoplasm)) OR TS=(Neoplasm, Bladder)) OR TS=(Urinary Bladder Cancer)) OR TS=(Cancer, Urinary Bladder)) OR TS=(Malignant Tumor of Urinary Bladder)) OR TS=(Cancer of the Bladder)) OR TS=(Bladder Cancer)) OR TS=(Bladder Cancers)) OR TS=(Cancer, Bladder)) OR TS=(Cancer of Bladder) | 170,926 results |
| 3 | ((((((((TS=(randomized controlled trial )) OR TS=(controlled clinical trial )) OR TS=(Randomized)) OR TS=(placebo )) OR TS=(clinical trials as topic )) OR TS=(randomly )) OR TS=(Trial)) OR TS=(Prospective )) OR TS=(Retrospective) | 5,759,187  results |
| 4 | TS=(Neoadjuvant) | 89,125 results |
| 5 | 1 AND 2 AND 3 AND 4 | 224 results |

| Embase | | |
| --- | --- | --- |
| No. | Query | Results |
| 1 | 'pd-1 inhibitors':ab,ti OR 'pd 1 inhibitors':ab,ti OR 'pd-1 inhibitor':ab,ti OR 'inhibitor, pd-1':ab,ti OR 'pd 1 inhibitor':ab,ti OR 'programmed cell death protein 1 inhibitors':ab,ti OR 'programmed cell death protein 1 inhibitor':ab,ti OR pembrolizumab:ab,ti OR 'sch 900475':ab,ti OR lambrolizumab:ab,ti OR 'mk 3475':ab,ti OR keytruda:ab,ti OR nivolumab:ab,ti OR opdivo:ab,ti OR 'ono 4538':ab,ti OR 'mdx 1106':ab,ti OR mdx1106:ab,ti OR 'bms 936558':ab,ti OR bms936558:ab,ti OR toripalimab:ab,ti OR 'bgb a317':ab,ti OR camrelizumab:ab,ti OR carrelizumab:ab,ti OR 'shr 1210':ab,ti OR 'gls 010':ab,ti OR cemiplimab:ab,ti OR regn2810:ab,ti OR sintilimab:ab,ti OR ibi308:ab,ti OR 'ibi 308':ab,ti OR zimberelimab:ab,ti OR prolgolimab:ab,ti OR dostarlimab:ab,ti OR jemperli:ab,ti OR 'dostarlimab gxly':ab,ti OR 'tsr 042':ab,ti OR gsk4057190:ab,ti OR 'pd-l1 inhibitors':ab,ti OR 'pd l1 inhibitors':ab,ti OR 'pd-l1 inhibitor':ab,ti OR 'pd l1 inhibitor':ab,ti OR 'programmed death-ligand 1 inhibitors':ab,ti OR 'programmed death ligand 1 inhibitors':ab,ti OR 'pd-1-pd-l1 blockade':ab,ti OR 'blockade, pd-1-pd-l1':ab,ti OR 'pd 1 pd l1 blockade':ab,ti OR atezolizumab:ab,ti OR 'anti pdl1':ab,ti OR ('immunoglobulin g1, anti-':ab,ti AND 'human cd antigen cd274':ab,ti AND 'human monoclonal mdpl3280a heavy chain':ab,ti AND ', disulfide with human monoclonal mdpl3280a kappa-chain, dimer':ab,ti) OR mpdl3280a:ab,ti OR 'mpdl 3280a':ab,ti OR tecentriq:ab,ti OR rg7446:ab,ti OR 'rg 7446':ab,ti OR durvalumab:ab,ti OR medi4736:ab,ti OR 'medi 4736':ab,ti OR imfinzi:ab,ti OR avelumab:ab,ti OR 'msb 0010682':ab,ti OR msb0010682:ab,ti OR bavencio:ab,ti OR msb0010718c:ab,ti OR 'msb 0010718c':ab,ti | 46,248 results |
| 2 | 'urinary bladder neoplasms':ab,ti OR 'neoplasm, urinary bladder':ab,ti OR 'urinary bladder neoplasm':ab,ti OR 'bladder tumors':ab,ti OR 'bladder tumor':ab,ti OR 'tumor, bladder':ab,ti OR 'tumors, bladder':ab,ti OR 'neoplasms, bladder':ab,ti OR 'bladder neoplasms':ab,ti OR 'neoplasm, bladder':ab,ti OR 'urinary bladder cancer':ab,ti OR 'cancer, urinary bladder':ab,ti OR 'malignant tumor of urinary bladder':ab,ti OR 'cancer of the bladder':ab,ti OR 'bladder cancer':ab,ti OR 'bladder cancers':ab,ti OR 'cancer, bladder':ab,ti OR 'cancer of bladder':ab,ti | 70,850 results |
| 3 | 'randomized controlled trial':ab,ti OR 'controlled clinical trial':ab,ti OR randomized:ab,ti OR placebo:ab,ti OR 'clinical trials as topic':ab,ti OR randomly:ab,ti OR trial:ab,ti OR prospective:ab,ti OR retrospective:ab,ti | 4,077,947 results |
| 4 | neoadjuvant:ab,ti | 90,327 results |
| 5 | 1 AND 2 AND 3 AND 4 | 174 results |

| cochrane library | | |
| --- | --- | --- |
| No. | Query | Results |
| 1 | (PD-1Inhibitors OR PD-1 Inhibitors OR PD 1 Inhibitors OR PD-1 Inhibitor OR Inhibitor, PD-1 OR PD 1 Inhibitor OR Programmed Cell Death Protein 1 Inhibitor OR Programmed Cell Death Protein 1 Inhibitors OR Nivolumab OR Opdivo OR ONO-4538 OR ONO 4538 OR ONO4538 OR MDX-1106 OR MDX 1106 OR MDX1106 OR BMS-936558 OR BMS 936558 OR BMS936558 OR Pembrolizumab OR SCH-900475 OR lambrolizumab OR MK-3475 OR Keytruda OR Tislelizumab OR BGB-A317 OR Toripalimab OR Camrelizumab OR carrelizumab OR SHR-1210 OR SHR 1210 OR Sintilimab OR IBI 308 OR IBI308 OR IBI-308 OR Zimberelimab OR GLS-010 OR Prolgolimab OR Dostarlimab OR Jemperli OR dostarlimab-gxly OR TSR-042 OR GSK4057190 OR PD-L1 Inhibitors OR PD-L1 Inhibitors OR PD L1 Inhibitors OR PD-L1 Inhibitor OR PD L1 Inhibitor OR Programmed Death-Ligand 1 Inhibitors OR Programmed Death Ligand 1 Inhibitors OR PD-1 PD-L1 Blockade OR Blockade, PD-1 PD-L1 OR PD 1 PD L1 Blockade OR Atezolizumab OR anti-PDL1 OR immunoglobulin G1, anti-(human CD antigen CD274) (human monoclonal MDPL3280a heavy chain), disulfide with human monoclonal MDPL3280a kappa-chain, dimer OR MPDL3280A OR MPDL-3280A OR Tecentriq OR RG7446 OR RG-7446 OR Durvalumab OR MEDI4736 OR MEDI-4736 OR Imfinzi OR Avelumab OR MSB-0010682 OR MSB0010718C OR MSB-0010718C OR MSB0010682 OR bavencio):ti,ab,kw | 13,213 results |
| 2 | (Urinary Bladder Neoplasms OR Neoplasm, Urinary Bladder OR Urinary Bladder Neoplasm Bladder Tumors OR Bladder Tumor OR Tumor, Bladder OR Tumors, Bladder OR Neoplasms, Bladder OR Bladder Neoplasms OR Bladder Neoplasm OR Neoplasm, Bladder OR Urinary Bladder Cancer OR Cancer, Urinary Bladder OR Malignant Tumor of Urinary Bladder OR Cancer of the Bladder OR Bladder Cancer OR Bladder Cancers OR Cancer, Bladder OR Cancer of Bladder):ti,ab,kw | 6,402 results |
| 3 | (randomized controlled trial OR controlled clinical trial OR Randomized OR placebo OR clinical trials as topic OR randomly OR Trial OR Prospective OR Retrospective):ti,ab,kw | 1,505,240 results |
| 4 | (Neoadjuvant):ti,ab,kw | 11,573 results |
| 5 | #2 AND #3 AND #4 AND #5 | 115 results |
