# Supplementary material for: Efficacy and safety of neoadjuvant PD-1 inhibitors or PD-L1 inhibitors for muscle invasive bladder cancer: a systematic review and meta-analysis
Source: Front Immunol. 2024 Jan 9;14:1332213. doi: 10.3389/fimmu.2023.1332213 (PMC10803485; doi:10.3389/fimmu.2023.1332213)
Supplement: Supplementary file 2 [file Table_2.doc]

Oncological and safety outcomes.

| Author Study arm(s) | | pCR, n pPR (%) |  | ≥ Grade ≥ Grade 3 surgical 3 irAEs complications | Steroid requirement | Tx- related death |
| --- | --- | --- | --- | --- | --- | --- |
| Szabados Atezolizumab | 87 | 27 |  | 10 16 |  | 1 |
|  |  | (31.0%) |  |  |  |  |
| Koshkin Atezolizumab | 20 | 2 (10.0%) 5 |  | 2 |  |  |
| Wei Durvalumab | 8 | 1 (12.5%) 2 |  | 1 |  |  |
| Goubet Pembrolizumab | 34 | 10 |  |  |  |  |
|  |  | (29.4%) |  |  |  |  |
| Basile Pembrolizumab | 112 | 42 63 | 1 PD | 8 25 | 4 |  |
|  |  | (37.5%) | 7 SD |  |  |  |
| Guercio armA: Nivolumab | armA: | armA: 2 4 | armA: 2 PD | 1 |  |  |
| armB: Nivolumab + | 11 | (18.2%) 3 | armB: 3 PD | 4 |  |  |
| Ipilimumab | armB: 9 | armB: 1  (11.1%) |  |  |  |  |
| Yin armA: Nivolumab | armA: | armA: 1 armA: | armA: 1 PD | armA: 0 |  |  |
| armB: Nivolumab +  lirilumab | 12  armB:29 | (8.3%) 2  armB: 5 armB: |  | armB: 4 |  |  |
|  |  | (17.2%) 8 |  |  |  |  |
| Van Dijk Nivolumab + | 24 | 11 14 |  | 13 |  |  |
| Ipilimumab |  | (45.8%) |  |  |  |  |
| Van Dorp Nivolumab +  Ipilimumab | 26 | 7 (26.9%) 11 | 1 PD |  |  |  |
| Kim GC+ Nivolumab | 34 | 12 22 |  |  |  |  |
|  |  | (35.3%) |  |  |  |  |
| Gupta GC+ Nivolumab | 39 | 20 27 |  | 3 | 0 |  |
|  |  | (51.3%) |  |  |  |  |
| Funt GC+ Atezolizumab | 39 | 16 27 | 2 PD | 5 | 2 |  |
|  |  | (41.0%) |  |  |  |  |
| Xing GC+ Camrelizumab | 11 | 6 (54.5%) 7 | 1 PD | 0 |  |  |
| Rose GC+ Pembrolizumab | 38 | 14 22 |  |  |  |  |
|  |  | (36.8%) |  |  |  |  |
| Grande armA: Durvalumab+  Tremelimumab | armA: 20 | armA: 8  (40.0%) | armA: 1 PD  armB: 2 PD | armA: 5  armB: 18 |  |  |
| armB: GC/ddMVAC | armB: | armB: 19 |  |  |  |  |
|  | 35 | (54.3%) |  |  |  |  |
| Gao Durvalumab+ | 24 | 9 (37.5%) 14 | 5 PD, 2 SD | 6 | 4 |  |
| Tremelimumab |  |  |  |  |  |  |
| Kaimakliotis GC+ Pembrolizumab | 36 | 16 22 |  |  |  |  |
|  |  | (44.4%) |  |  |  |  |
| Cathomas GC+ Durvalumab | 53 | 18 32 | 1 PD | 16 |  |  |
|  |  | (34.0%) |  |  |  |  |
| Thibault ddMVAC+ Durvalumab  ± Tremelimumab | 12 | 8 (66.7%) 9 |  | 0 |  |  |
| Hristos Gemcitabine+ | 34 | 18 19 | 3 PD | 4 |  |  |
| Pembrolizumab |  | (52.9%) |  |  |  |  |
| Chanza armA: PG+ Avelumab | armA: | armA: 5 6 | armA: 1 PD | armA: 2 |  |  |
| armB: Avelumab | 27 | (18.5%) 11 |  |  |  |  |
|  | armB: 28 | armB: 10  (35.7%) |  |  |  |  |
| Lin CG+ Tislelizumab | 17 | 10 13 |  |  |  |  |
|  |  | (58.8%) |  |  |  |  |

AEs reported in included studies

|  |  | Number of reported studies | Events | Morbidity |
| --- | --- | --- | --- | --- |
| ≥ Grade 3 irAEs | Liver enzymes increase | 5 | 14 | 4.7% |
| (PD-(L)1 inhibitors plus chemotherapy) | Amylase/lipase increase | 4 | 13 | 4.3% |
|  | imDC | 3 | 7 | 2.3% |
|  | Hematological toxicity | 3 | 4 | 1.3% |
|  | Skin reaction | 2 | 3 | 1.0% |
|  | Electrolyte disorder | 2 | 3 | 1.0% |
|  | Neuropathy | 2 | 2 | 0.7% |
|  | Fatigue | 2 | 2 | 0.7% |
|  | Pneumonitis | 2 | 2 | 0.7% |
|  | Adenitis | 1 | 2 | 0.7% |
|  | Xerostomia/Sjögren syndrome | 1 | 1 | 0.3% |
|  | Myocarditis | 1 | 1 | 0.3% |
|  | Hyperglycemia | 1 | 1 | 0.3% |
| ≥ Grade 3 TRAEs | Hematological disorders |  | 19 | 38.8% |
| (Chemoimmunotherapy only) Fatigue | |  | 2 | 4.1% |
| Anal abscess | |  | 1 | 2.0% |
| Renal insufﬁciency | |  | 1 | 2.0% |
